# Engineered circular guide RNAs boost CRISPR/Cas12a- and CRISPR/Cas13d-based DNA and RNA editing

**DOI:** 10.1186/s13059-023-02992-z

**Published:** 2023-06-23

**Authors:** Xin Zhang, Xinlong Wang, Jie Lv, Hongxin Huang, Jiahong Wang, Ma Zhuo, Zhihong Tan, Guanjie Huang, Jiawei Liu, Yuchen Liu, Mengrao Li, Qixiao Lin, Lian Li, Shufeng Ma, Tao Huang, Ying Lin, Xiaoyang Zhao, Zhili Rong

**Affiliations:** 1 Dongguan Institute of Clinical Cancer Research, Affiliated Dongguan Hospital, Southern Medical University, Dongguan, 523058 China; 2 Cancer Research Institute, School of Basic Medical Sciences, State Key Laboratory of Organ Failure Research, National Clinical Research Center of Kidney Disease, Key Laboratory of Organ Failure Research (Ministry of Education), Southern Medical University, Guangzhou, 510515 China; 3 Dermatology Hospital, Southern Medical University, Guangzhou, 510091 China; 4 Department of Nephrology, Shenzhen Hospital, Southern Medical University, Shenzhen, 518110 China; 5 Department of Development, School of Basic Medical Sciences, State Key Laboratory of Organ Failure Research, National Clinical Research Center of Kidney Disease, Key Laboratory of Organ Failure Research (Ministry of Education), Southern Medical University, Guangzhou, 510515 China

**Keywords:** cgRNA, Engineered circular gRNA, Cas12a, Cas13d, Gene activation, DNA editing, RNA editing

## Abstract

**Background:**

The CRISPR/Cas12a and CRISPR/Cas13d systems are widely used for fundamental research and hold great potential for future clinical applications. However, the short half-life of guide RNAs (gRNAs), particularly free gRNAs without Cas nuclease binding, limits their editing efficiency and durability.

**Results:**

Here, we engineer circular free gRNAs (cgRNAs) to increase their stability, and thus availability for Cas12a and Cas13d processing and loading, to boost editing. cgRNAs increases the efficiency of Cas12a-based transcription activators and genomic DNA cleavage by approximately 2.1- to 40.2-fold for single gene editing and 1.7- to 2.1-fold for multiplexed gene editing than their linear counterparts, without compromising specificity, across multiple sites and cell lines. Similarly, the RNA interference efficiency of Cas13d is increased by around 1.8-fold. In in vivo mouse liver, cgRNAs are more potent in activating gene expression and cleaving genomic DNA.

**Conclusions:**

CgRNAs enable more efficient programmable DNA and RNA editing for Cas12a and Cas13d with broad applicability for fundamental research and gene therapy.

**Supplementary Information:**

The online version contains supplementary material available at 10.1186/s13059-023-02992-z.

## Background

The CRISPR-Cas nucleases are widely used for DNA and RNA editing in human and other cells and organisms and have broad applications in fundamental biological research and translational medicine [1, 2]. Three types of Cas nucleases are most commonly used: Cas9, Cas12a (formerly Cpf1), and Cas13, exemplified by *Streptococcus pyogenes* Cas9 (*Sp*Cas9) [3, 4], *Acidaminococcus sp.* Cas12a (*As*Cas12a) and *Lachnospiraceae bacterium ND2006* Cas12a (*Lb*Cas12a) [5, 6], and *Ruminococcus flavefaciens XPD3002* Cas13d (*Rfx*Cas13d, also known as CasRx) [7, 8], respectively.

Both Cas9 and Cas12a edit DNA. Different from Cas9, Cas12a exhibits several unique features. First, Cas12a exhibits higher specificity than Cas9, which enables more precise gene editing for therapeutic applications [9–11]. Second, Cas12a possesses crRNA self-processing capability [5, 12], enabling multiplexed gene editing with a single gRNA transcript [13–15], which is also a feature of Cas13 [7]. Third, rather than a G-rich protospacer adjacent motif (PAM) for Cas9, Cas12a recognizes a T-rich PAM, which makes it a good complement to Cas9 and thus broadens the genomic targeting scope [5]. Forth, Cas12a cuts target DNA with a single RuvC domain to generate sticky ends instead of with both RuvC and HNH domains for Cas9 to generate blunt ends [5, 16]. Finally, Cas12a is able to trans-cleave single-strand DNA, making it a powerful tool for nucleic acid detection [17, 18].

From the point of specificity, Cas12a-based DNA editing induces less off-target effect with safety risk than Cas9 when applied for genomic DNA sequence change, gene activation, or gene repression. As well, Cas13-based RNA editing is generally reversible and tunable without causing permanent genomic DNA changes. Therefore, Cas12a and Cas13 may hold certain advantages over Cas9 in therapeutic applications. However, relatively low editing efficiency hinders fulfilling their promise of therapeutic editing.

Both Cas nuclease and gRNA may affect editing efficiency. For example, Cas12a engineering is an effective strategy to enhance activity, like the engineered variants, Cas12a-Plus, en*As*Cas12a, *As*Cas12a-HF, *As*Cas12a-Ultra, and hyperCas12a [19–21]. However, it is believed that gRNA may play a more important role to limit editing efficiency because RNA is well known for its high sensitivity to be rapidly degraded by endonucleases and exonucleases. As proof, it has been reported that gRNAs especially free gRNAs (without Cas protein binding) are extremely unstable [22]. Chemical modification at the gRNA ends to reduce degradation by exonucleases is able to significantly enhance gene editing both in vitro and in vivo [23–25]. Therefore, it is essential to increase the stability and thus the abundance of gRNAs for efficient processing and loading by Cas12a and Cas13 to enhance editing efficiency.

Circular RNA is a highly stable RNA species because its covalently closed ring structure is resistant to degradation by exonucleases [26–30]. And circular ADAR-recruiting RNAs have been recently reported to increase the efficiency of A-to-I RNA editing [31, 32]. Therefore, we utilized circular free gRNAs and the gRNA self-processing ability of Cas12a and CasRx to increase the efficiency of Cas12a-based transcription activators as well as the DNA and RNA cleavage efficiency of Cas12a and CasRx.

## Results

### Stabilization of gRNA by circularization in human cells

**Fig. 1:**
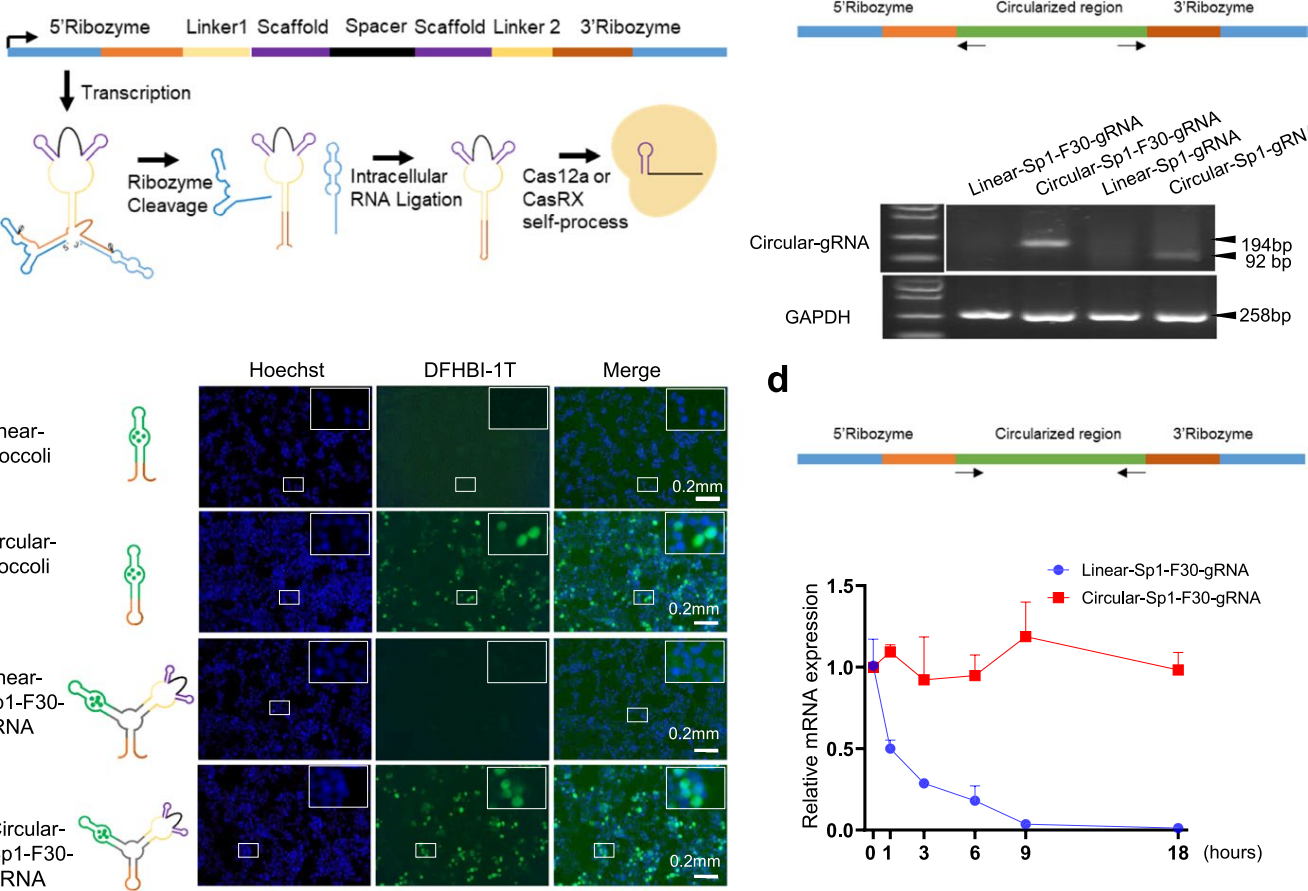
Circularization increases the stability of gRNAs in human cells.** a** Schematic of circular guide RNAs (cgRNAs). **b** Broccoli fluorescence revealed the abundance of circular RNAs in cells. HEK293T cells were transfected with indicated plasmids encoding linear or circular RNAs and live stained with DFHBI-1T 48 hours (hrs) after transfection. **c** Reverse transcription PCR (RT-PCR) revealed circularization of RNAs in cells. HEK293T cells were transfected with indicated plasmids encoding linear or circular RNAs, and RNA was harvested 72 hrs after transfection, followed by RT-PCR with indicated outward-facing primers. **d** Stability of circular gRNA in cells. HEK293T cells were treated with actinomycin D for 1, 3, 6, 9, 18 hrs starting at 24 hrs post-transfection with plasmids encoding linear or circular RNAs, and RNA was harvested for quantitative RT-PCR analysis. *n* = 3 independent experiments

To circularize gRNAs in mammalian cells, we adopted the elegant Tornado expression system [29]. As shown in Fig. 1a, we flanked the gRNAs by Twister ribozymes, which undergo autocatalytic cleavage, leaving termini to be ligated by the ubiquitous endogenous RNA ligase RtcB to yield circular gRNAs (cgRNAs). With gRNA self-processing ability, Cas12a and CasRx then cleaved and loaded the cgRNAs. Via mFold prediction [33], the Sp1 linker was designed to maintain the gRNA structure to be correctly recognized and processed by Cas nucleases. To visualize the expression of gRNAs, the Broccoli RNA aptamer was integrated into the circular RNA via an F30 3-way junction, which could bind the fluorophore DFHBI-1T and activate green fluorescence [29, 34]. Live staining of transfected HEK293T cells showed that circular RNAs were more abundant than their linear counterparts (Fig. 1b). Reverse transcription PCR using outward-facing primers which selectively amplified only the circular gRNAs demonstrated that gRNAs were circularized in cells (Fig. 1c). In addition, using actinomycin D treatment to block RNA transcription, we checked the stability of the circular gRNA and the linear counterparts and found that the former is much more stable than the latter (Fig. 1d). Therefore, all the above data demonstrated that circularization increased the stability of gRNAs in human cells.

### Circular gRNAs promote Cas12a-based transcriptional activation

**Fig. 2:**
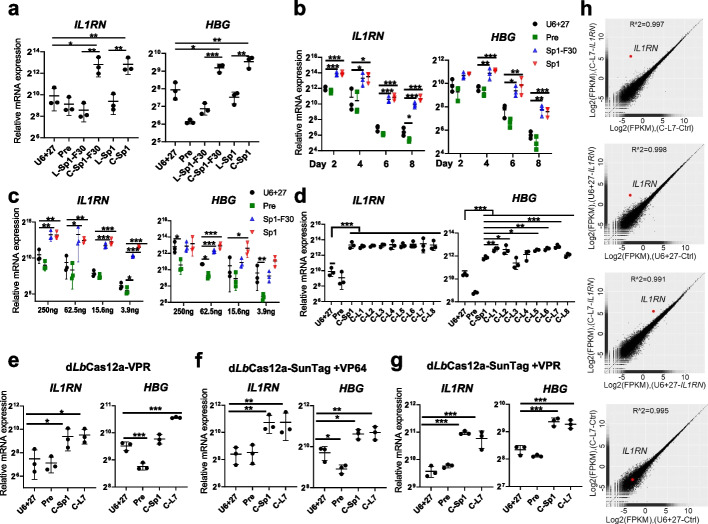
Circular guide RNAs increase the transcription efficiency of Cas12a-based activators.** a** cgRNA-directed gene activation in a d*Lb*Cas12a-p300 knock-in (KI) HEK293T cell line. **b** Time-course analyses of cgRNA-directed gene activation in the KI cells. **c** Dose-dependent analyses of cgRNA-directed gene activation in the d*Lb*Cas12a-p300 knock-in HEK293T cells.** d** Gene activation guided by cgRNAs with different linkers in the KI cells. **e–g** cgRNA-directed gene activation with a variant of d*Lb*Cas12a-based gene activators in HEK293T cells transiently co-transfected with indicated activator-encoded and gRNA-encoded plasmids. **h** The specificity of cgRNA-directed gene activation. Gene expression plot generated from RNA-seq data from the KI HEK293T cells transfected with U6 + 27 linear gRNAs or C-L7 cgRNAs targeting mNeonGreen (control) or *IL1RN*. R indicates Pearson’s correlation coefficient. The average of three biological replicates was shown. For **a–g**, quantitative RT-PCR revealed relative mRNA expression of *IL1RN* and *HBG*. Mean values are presented with S.D., *n* = 3 independent experiments. For each experiment, fold changes of mRNA expression in tested samples versus that in the U6 + 27 linear mNeonGreen gRNA were shown. *p <0.05, **p <0.01, ***p <0.001, one-way ANOVA test

To test whether cgRNA could enhance Cas nuclease-based editing, we first analyzed the transcription efficiency of dCas12a-based gene activators. In a doxycycline-inducible d*Lb*Cas12a-p300 knock-in (KI) HEK293T cell line (Additional file 1: Fig. S1), transiently transfected plasmids encoding cgRNAs (C-Sp1-F30 and C-Sp1) activated *IL1RN* and *HBG* gene expression more potently than the linear counterparts (L-Sp1-F30 and L-Sp1), as well as commonly used mature gRNAs (U6 + 27) and unprocessed gRNAs (Pre, spacer flanked by two scaffold sequences) (Fig. 2a). All the gRNAs were driven by the polymerase III promoter U6 + 27 cassette, which has been reported to improve the stability of small interfering RNA [35]. Time-course and dose-dependent analyses showed that cgRNAs exhibited better durability and performance than U6 + 27 and Pre gRNAs (Fig. 2b, c). Because linker sequences were essential to maintain the structure and function of cgRNAs, we optimized the length and component of linker sequences. Through screening several digital libraries generated by random sequences with RNAfold and mFold prediction [33, 36], 8 linkers were selected out for the wet-experiment test, and C-linker7 (C-L7) was found to be the best one, which was improved 5.5–12.2-fold changes than U6+27 (Fig. 2d). As expected, about 3.3–5.7-fold changes increased efficiency was also observed in MCF7 cells (Additional file 1: Fig. S2a). We further demonstrated that cgRNAs were applicable for other d*Lb*Cas12a-based activators, including d*Lb*Cas12a-VPR, -SunTag-VP64, and -SunTag-VPR (Fig. 2e–g; Additional file 1: Fig. S2b). Finally, RNA-seq analyses showed that only the *IL1RN* target gene was significantly activated, indicating a high specificity for cgRNA-directed gene activation (Fig. 2h; Additional file 1: Fig. S2c). In summary, cgRNAs could enhance the activity and maintain the high specificity of Cas12a-based gene activators.

### Circular gRNAs increase the DNA cleavage efficiency of Cas12a

**Fig. 3:**
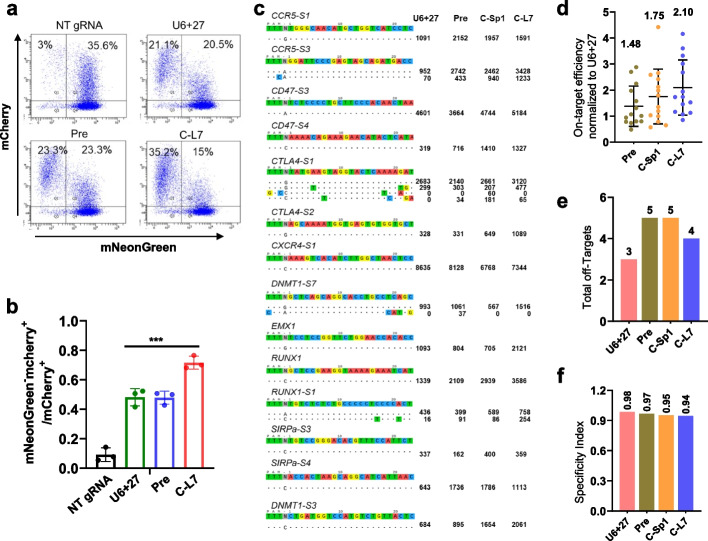
Circular guide RNAs increase the DNA cleavage efficiency of Cas12a.** a** Fluorescence-activated cell sorting (FACS) analyses of the mNeonGreen reporter cells 4 days after co-transfection with *Lb*Cas12a-P2A-mCherry and mNeonGreen-targeting-gRNA plasmids. **b** The cleavage efficiency was quantified by the cell ratio of mNeonGreen^‒^ mCherry^+^ / mCherry^+^ in the FACS assays. *n* = 3 independent experiments. NT gRNA, non-targeting gRNA, which recognized no site in the human genome and transcriptome. ***p <0.001, one-way ANOVA test. **c–f** The efficiency and specificity of different gRNAs-directed DNA cleavage at 14 sites in a *Lb*Cas12a knock-in HEK293T cell line revealed by Tag-seq. The gRNA reference as well as the on-target and off-target sites was shown on the left, and sequencing read counts were shown to the right of each site (**c**). Efficiency comparison between different gRNAs (**d**). The total number of off-target sites detected for the 14 sites (**e**). Specificity index (value was calculated by the ratio of total on-target reads to the on-target reads plus the off-target reads within the 14 sites) (**f**)

Next, we tested whether cgRNA could enhance Cas12a-mediated DNA cleavage. We designed different forms of gRNAs targeting the same site within the mNeonGreen gene and co-transfected these plasmids with *Lb*Cas12a-mCherry into a mNeonGreen reporter HEK293T cell line. Fluorescence-activated cell sorting (FACS) analyses showed that C-L7 for *Lb*Cas12a significantly enhanced DNA editing activity, with about 1.4- and 1.5-fold than U6+27 and Pre gRNA (Fig. 3a, b; Additional file 1: Fig. S3a). Next, using Tag-seq [37] and a doxycycline-induced *Lb*Cas12a knock-in HEK293T cell line (Additional file 1: Fig. S3b-d), transiently transfected plasmids encoding cgRNAs showed higher efficiency and slight lower specificity compared with linear U6 + 27 gRNAs across 14 genomic sites (Fig. 3c–f; Additional file 1: Fig. S3e), with about 1.75- and 2.10-fold for C-Sp1 and C-L7 in efficiency, respectively (Fig. 3c, d), and with 3, 5, and 4 off-target sites as well as specificity index (the ratio of total on-target reads to the on-target reads plus the off-target reads) of 0.98, 0.95, and 0.94 for U6 + 27, C-Sp1, and C-L7, respectively (Fig. 3e, f; Additional file 1: Fig. S3e). Similar results were observed in MCF7 cells (Additional file 1: Fig. S4).

Next, we performed comparison between cgRNA of other extended structuralized gRNAs. According to reports, extending the 5′ end of the crRNA by 9 nucleotides (gRNA + 9) and 59 nucleotides (gRNA + 59) could enhance the gene editing efficiency of the Cas12a ribonucleoproteins complexes (RNP) [38]. And the crRNA with a U_4_AU_4_ 3′-overhang was more favorable binding to Cas12a to improve the activity [39]. In the doxycycline-inducible d*Lb*Cas12a-p300 knock-in HEK293T cell line, transiently transfected plasmids encoding gRNAs, cgRNAs (C-L7-d27, without 27 nucleotides at the 5′ end, and C-L7) showed more potently activation than the 5′ end extended gRNA (gRNA + 9 and gRNA + 59) with about 8.8–11.4-fold change in gene *IL1RN* and 4.9–6.3-fold change in gene *HBG*, as well as 3′ end extended gRNA (gRNA + T_4_AT_4_) with about 4.0–7.6-fold change in gene *IL1RN* and 6.5–9.1-fold change in gene *HBG* (Additional file 1: Fig. S5a). Moreover, we tested DNA cleavage and found that cgRNA (C-L7-d27 and C-L7) performed 1.6–1.9-fold change activity than 5′ end extended gRNA (+ 9 and + 59) and 1.5–1.7-fold change activity than 3′ end extended gRNA (gRNA + T_4_AT_4_) to disrupt mNeonGreen in the HEK293T reporter cell line (Additional file 1: Fig. S5b, c). Likewise, cgRNA (C-L7-d27 and C-L7) performed about 1.6–2.0-fold change activity than 5′ end extended gRNA (+ 9 and + 59) and about 1.3–1.5-fold change activity than 3′ end extended gRNA (gRNA + T_4_AT_4_) to disrupt endogenous gene *VEGFA* in the doxycycline-inducible *Lb*Cas12a knock-in HEK293T cell line (Additional file 1: Fig. S5d, e).

*As*Cas12a is another popular Cas12a nuclease and thus we tested whether cgRNAs were applicable to it. The transcriptional activator d*As*Cas12a-VPR activated *IL1RN* and *HBG* more efficiently when coupled with cgRNAs, including C-Sp1-F30, C-Sp1, and 4 cgRNAs with different linkers which were screened out of digital libraries via RNAfold and mFold prediction (Additional file 1: Fig. S6a, b). Since the cgRNA C-L1 performed best among all the tested 6 cgRNAs for gene activation, we tested its performance in DNA cleavage and found that C-L1 outperformed the counterpart linear gRNAs to disrupt mNeonGreen in the HEK293T reporter cell line (Additional file 1: Fig. S6c-e).

### Circular gRNAs improve Cas12a-mediated multiplexed gene activation and DNA cleavage

Multiplexed editing with a single gRNA transcript is a unique feature of Cas12a over Cas9; therefore, we tested whether cgRNAs were compatible or even improved multiplexed editing. The gRNAs targeting the promoter regions of *NTT*, *IL1RN*, and *HBG* were co-expressed within a single linear (Pre-NIH) or circular (C-L7-NIH) transcript and C-L7-NIH significantly improved d*Lb*Cas12a-VPR-mediated transcriptional activation of each of the three genes (Additional file 1: Fig. S7a). Similarly, Deep-seq revealed that the circular transcript (C-L7-CVDER) containing 5 gRNAs targeting *CD47*-S3, *VEGFA*-S1, *DNMT1*-S3, *EMX1*, and *RUNX1* was more potent to direct *Lb*Cas12a-mediated DNA cleavage than the linear transcript (Pre-CVDER, Additional file 1: Fig. S7b). Tag-seq also showed that C-L7-CVDER exhibited a 1.67-fold efficiency and slight lower specificity when compared to Pre-CVDER (Additional file 1: Fig. S7c-g).

In summary, all the above results demonstrated that circular gRNAs were able to increase the efficiency and maintain almost equal specificity of *Lb*Cas12a- and *As*Cas12a-based gene activation and DNA cleavage.

### Circular gRNAs increase the RNA cleavage efficiency of CasRx

**Fig. 4:**
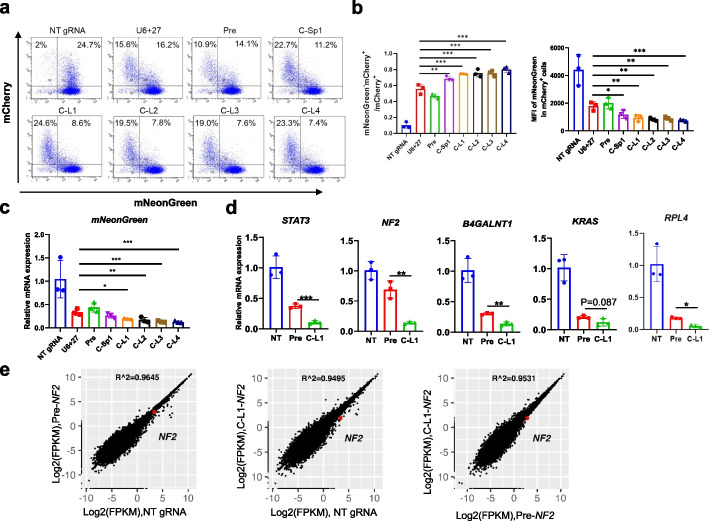
Circular guide RNAs increase the RNA cleavage efficiency of CasRx.** a, b** The RNA cleavage efficiency of *Rfx*Cas13d (CasRx) on the mNeonGreen reporter gene. FACS analyses of the mNeonGreen knock-in HEK293T cell line 48 hrs after co-transfection with CasRx-P2A-mCherry and mNeonGreen-targeting-gRNA plasmids (**a**). The cleavage efficiency was quantified by the cell ratio of mNeonGreen^‒^ mCherry^+^ / mCherry^+^ and mean fluorescence intensity (MFI) of mNeonGreen in transfected cells (mCherry positive) in the FACS assays (**b**). **c** Relative degradation of mNeonGreen transcripts induced by CasRx with circular or linear gRNAs. The mRNA expression levels were determined by RT-PCR. **d** The RNA cleavage efficiency of CasRx on endogenous genes. HEK293T cells were co-transfected with CasRx-P2A-mCherry and gRNA plasmids, and mCherry^+^ cells were sorted out by FACS for RNA extraction and quantitative RT-PCR analyses. *n* = 3 independent experiments. NT, Non-targeting gRNA, which recognized no site in the human genome and transcriptome. **e** The specificity of cgRNA-directed gene degradation. Gene expression plot generated from RNA-seq data from HEK293T cells transfected with non-targeting cgRNA or Pre linear gRNAs or C-L1 cgRNAs targeting *NF2*. R indicates Pearson’s correlation coefficient. The average of three biological replicates was shown. For **b**,**c**, **p <0.01, ***p <0.001, one-way ANOVA test. For **d**, **p <0.01, ***p <0.001, Student’s *t* test

Further, we tested whether cgRNAs could enhance CasRx-mediated RNA cleavage. To this end, we selected 4 circular RNA backbones with different linkers out of digital libraries via RNAfold and mFold prediction and did similar experiments in the mNeonGreen reporter cell line. FACS analyses and reverse transcription PCR (RT-PCR) showed that cgRNAs significantly increased RNA cleavage efficiency with about 1.2–1.4-fold change (Fig. 4a–c; Additional file 1: Fig. S8a). For endogenous genes (*STAT3*, *NF2*, *B4GALNT1*, *KRAS*, and *RPL4*) in HEK293T cells, C-L1 cgRNAs were also observed to repress their expression more potently than the linear counterpart Pre gRNAs with about 1.2–2.8-fold change (Fig. 4d). Similar results were observed in MCF7 cells (Additional file 1: Fig. S8b). Moreover, RNA-seq analyses showed that *NF2* (the target gene) was significantly decreased in the C-L1 cgRNA sample compared to the Pre and NT gRNA samples with 2.4-fold and 3.6-fold change, respectively (Fig. 4e; Additional file 1: Fig. S8c). Of note, 64 and 126 differentially expressed genes (DEGs) were observed when comparing the C-L1 cgRNA group to the NT gRNA group and to the Pre gRNA group, respectively, indicating nonspecific and collateral cutting as well as the associated secondary effects [40, 41].

Collateral effect is a feature of Cas13 protein which may hindered their application in vivo [41]. To evaluate the effect, we first tested the trans-cleavage activity (cleavage of mCherry) of CasRx when targeting the exogenously expressed mNeonGreen gene. As expected, cgRNAs showed significantly decreased mNeonGreen fluorescence intensity, and with a similar trans-cleavage activity compared with linear U6 +27 gRNA (Additional file 1: Fig. S9). Next, we tested the trans-cleavage activity of CasRx when targeting endogenous genes. Consistent with the previous report [40], *RPL4* gRNA induced dramatic collateral effects. However, only slight collateral degradation was observed when targeting *STAT3* and *NF2*, and no apparent collateral degradation was observed for *B4GALNT1* and *KRAS*. More importantly, we observed no significant difference between cgRNAs and linear Pre gRNA (Additional file 1: Fig. S10).

### Circular gRNAs enhance Cas12a-based transcriptional activation in vivo

**Fig. 5:**
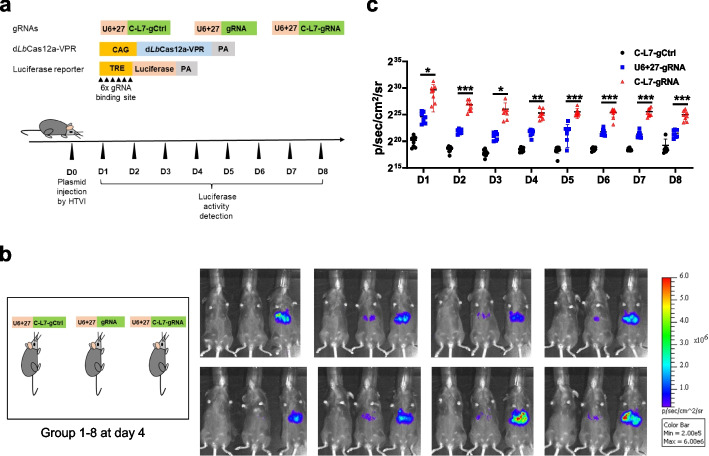
Circular gRNAs enhance the activation efficiency of d*Lb*Cas12a-VPR in vivo***. a*** Experiment design for d*Lb*Cas12a-VPR to activate Luciferase expression in mouse liver. gCtrl, control gRNA, which targeted mNeonGreen. HTVI, hydrodynamic tail vein injection. **b** Representative bioluminescence imaging results at day 4 for all the 8 groups of mice. **c** Quantification of bioluminescence imaging detected for a consecutive 8 days. *p <0.05, **p <0.01, ***p <0.001, Student’s *t* test

Next, we explored whether cgRNAs could improve gene activation in vivo. To this end, we constructed the TRE-Luciferase-pA plasmid, which contained 6 gRNA binding sites within the TRE promoter region. The TRE-Luciferase-pA plasmid and d*Lb*Cas12a-VPR, as well as a control cgRNA targeting mNeonGreen or a linear gRNA or a C-L7 cgRNA targeting the TRE promoter, were co-delivered to mouse liver via hydrodynamic tail vein injection (HTVI) [42], and from the next day, luciferase activity was live examined each day for a consecutive 8 days (Fig. 5a). Similar to the in vitro results, cgRNA outperformed linear gRNA at each time point with about 9.9–32.6-fold change (Fig. 5b, c).

### Circular gRNAs enhance the DNA cleavage efficiency of Cas12a in vivo

**Fig. 6:**
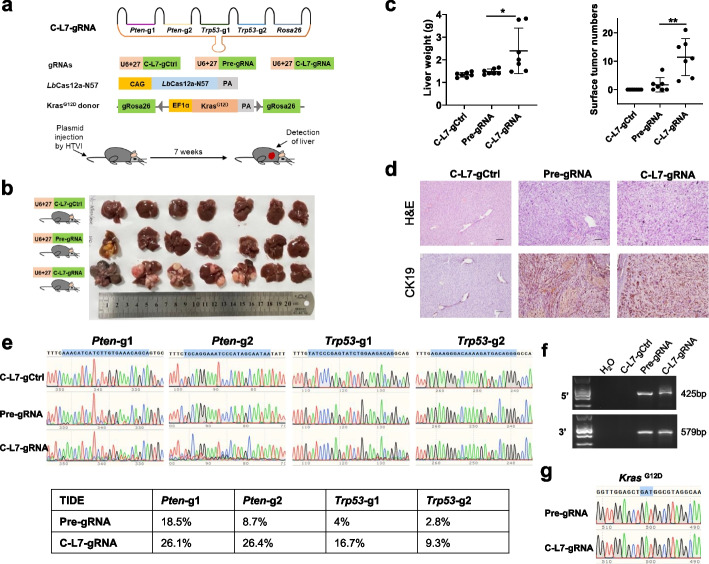
Circular gRNAs enhance the DNA cleavage efficiency of *Lb*Cas12a in vivo***. a*** Experiment design for *Lb*Cas12a-N57 to induce liver tumor in adult mice. gCtrl, control gRNA, which targeted mNeonGreen. HTVI, hydrodynamic tail vein injection. **b** Image analysis of mouse liver harvested 7 weeks after injection. **c** Quantification of liver weight and surface liver tumor nodules per mouse. *n* = 7. **d** Representative images of H&E and IHC staining of Ck19 in mouse liver tumors. Scale bar, 100 μm. **e** Sanger sequencing results and tracking of indels by decomposition (TIDE) analyses of tumor DNA for *Pten* and *Trp53* targeted sites. Blue shadow denoted the gRNA recognizing sites. **f** Genomic PCR of targeted integration of KRAS^G12D^ donor in tumors. Primers for the 5′-junction and 3′-junction were R26–5-F/R26–5-R and R26–3-F/R26–3-R, respectively.** g** Verification of the presence of the Kras^G12D^ mutation in the tumors by Sanger sequencing. *p <0.05, **p <0.01, Student’s *t* test

Finally, we explored whether cgRNAs could improve DNA cleavage in vivo. To this end, we adopted the Cas-N57 system developed by our group to induce tumorigenesis in mouse liver [42]. Because *KRAS*, *TP53*, and *PTEN* mutants are the major drivers of intrahepatic cholangiocarcinoma (ICC) [43], we used *Lb*Cas12a-N57 to simultaneously insert *Kras*^*G12D*^ into the *Rosa26* site and disrupt *Trp53* and *Pten* in the mouse liver. As shown in Fig. 6a, the *Kras*^*G12D*^ donor and *Lb*Cas12a-N57 plasmids, as well as the plasmid encoding a C-L7 circular multiplexed transcript or a Pre linear multiplexed transcript containing 2 gRNAs for *Pten*, 2 gRNAs for *Trp53*, and 1 gRNA for *Rosa26* or encoding a control cgRNA targeting mNeonGreen, were co-delivered to mouse liver via hydrodynamic tail vein injection and tumorigenesis was examined 7 weeks after injection. The C-L7 cgRNA group showed more tumor nodules and the liver weighed more (Fig. 6b, c). H&E and immunochemical staining of the tumor nodules showed pathological features of bile duct differentiation and expression of the ICC marker cytokeratin 19 (Fig. 6d). Sanger sequencing and Tracking of indels by decomposition (TIDE) analyses of genomic PCR amplicons from tumor nodules showed that the C-L7 cgRNA induced more indels than the linear Pre gRNA (Fig. 6e). In addition, Sanger sequencing also demonstrated targeted insertion of *Kras*^*G12D*^ into the *Rosa26* locus (Fig. 6f, g). Collectively, these results suggested that circular gRNA was more potent than linear gRNA to cleave genomic DNA in vivo.

## Discussion

In this study, using the Tornado expression system [29], we generated cgRNAs, which were much more stable than linear gRNAs. cgRNAs increased the transcription efficiency of d*Lb*Cas12a-based gene activators by up to ~40-fold, including d*Lb*Cas12a-p300, d*Lb*Cas12a-VPR, d*Lb*Cas12a-SunTag-VP64, and d*Lb*Cas12a-SunTag-VPR systems. Further, cgRNA increased the genomic DNA cleavage efficiency of *Lb*Cas12a by ~2-fold. Apart from single gene editing, multiplexed gene editing with a single gRNA transcript was also enhanced by cgRNAs. More importantly, enhanced activity did not compromise specificity. And the enhancement phenomenon was also observed with *As*Cas12a, another popular Cas12a nuclease. Similarly, the RNA interference efficiency of CasRx was increased by ~2-fold when directed by cgRNAs and similar collateral effect was maintained. And finally, in mouse liver, cgRNAs were more potent to activate gene expression and were able to enhance Cas12a-mediated *Kras*^*G12D*^ insertion and *Pten* and *Trp53* disruption to promote tumorigenesis.

Owing to the delivery challenge associated with in vivo therapeutic gene editing, target cells generally uptake much less nucleic acid or ribonucleoprotein (RNP) encoding the editing tools than the cultured cells for in vitro or ex vivo gene editing [44]. Therefore, developing editing tools with high efficiency is an essential approach to fulfill the therapeutic efficacy of in vivo gene editing, apart from improving the delivery method itself. In this study, we engineered cgRNAs to enhance the efficiency of Cas12a-based gene activation and genomic DNA cleavage. The cgRNA system performed well for cultured cells, achieving durable editing for at least 8 days and efficient editing with extremely low amount of cgRNAs (Fig. 2b, c). Encouraged by these results in vitro, we performed gene activation and gene disruption experiments in adult mouse liver and found that cgRNAs significantly outperformed the linear gRNA counterparts (Figs. 5 and 6). These observations indicated a great potential for cgRNAs to be used for therapeutic gene editing in vivo in the future.

Because the secondary structure of RNA is essential for both ribozyme self-cleavage for circularization and Cas12a and CasRx self-processing and loading, the linker 1 and linker 2 between the ribozymes and the gRNA need to be optimized to maintain the correct RNA structure (Fig. 1a). We adopted adenine- and cytosine-rich (AC-rich) RNA linkers as they have been widely used as flexible RNA linkers [30, 32]. To further increase the performance, we generated several digital cgRNA libraries with variant linkers and screened out cgRNAs with the predicted most stable correct structure via RNAfold and mFold prediction and verified by wet experiments (Figs. 2d and 4a, b, Additional file 1: Fig. S6b, Fig. S11-13, and Linker design in the “ Methods” section). Restricted by the server computing power, we only screened limited digital cgRNA libraries. It is likely to find out better cgRNA linkers to further enhance editing efficiency after more digital libraries were screened. Moreover, we would suggest confirming the cgRNA structure by RNAfold and mFold prediction when designing a particular gRNA since the spacer sequence might interfere with the RNA secondary structure.

With gRNA self-processing capability, Cas12a and Cas13 are theoretically unable to form protein/gRNA complex with circular gRNA, and circularization of free gRNA is thus a proper approach to boost their editing activity. Without gRNA self-processing capability, it would be hard for Cas9 to load circular gRNA or to execute efficient editing when bound to circular gRNA. Our preliminary data indicated that Cas9 and circular gRNA might form a complex to edit genes with less efficiency. Extensive optimization of the gRNA scaffold and circular RNA linker facilitated by AI-assisted RNA and protein structure prediction might solve this problem in the near future [45, 46].

## Conclusion

In summary, we engineered free circular gRNA to boost programmable DNA and RNA editing for Cas12a and CasRx, which might have broad applications for fundamental biological research and translational medicine.

## Methods

### Plasmid construction

For U6+27 gRNA-expressing constructs, the DNA sequences including the hU6+27 promoter, a gRNA scaffold, and a gRNA insert site were synthesized and cloned into the pBluSKM vector. For Pre gRNA-expressing constructs, a second gRNA scaffold was inserted downstream of the gRNA insert site in the U6+27 gRNA-expressing constructs. For linear Sp1-F30 gRNA-expressing constructs, the DNA sequences including a 5' ligation sequence, a 5' 42-nt spacer, an F30 3-way junction, a broccoli sequence, two gRNA scaffolds with a gRNA insert site, a 3' 41-nt spacer, and a 3' ligation sequence were synthesized and cloned into the pBluSKM vector with the hU6+27 promoter. For circular sp1-F30 gRNA-expressing constructs, a Twister P3 U2A ribozyme sequence and a Twister P1 ribozyme sequence were inserted to flank the linear sp1-F30 gRNA-expressing constructs. For linear and circular sp1 gRNA-expressing constructs, the F30 3-way junction and the broccoli sequences were deleted in the corresponding F30 plasmids. For U6 gRNA-expressing constructs, 27-nt in the 3′ end of U6+27 was deleted in the corresponding U6+27 plasmids. For C-L7-d27 gRNA-expressing constructs, 27-nt in the 3′ end of U6+27 was deleted in the corresponding C-L7-d27 plasmids. For +9 or +59 gRNA-expressing constructs, a 9-nt or 59-nt 5′ extended sequence was added by Gibson Assembly in the corresponding U6 plasmids. For + T_4_AT_4_ gRNA-expressing constructs, the 3′ extended sequence (TTTTATTTT) was added by Gibson Assembly in the corresponding U6 plasmids.

For d*Lb*Cas12a-p300 and *Lb*Cas12a knock-in plasmids, the sequences of d*Lb*Cas12a-p300 and *Lb*Cas12a were amplified from the pCAG-d*Lb*Cas12a-p300-mCherry and the pCAG-*Lb*Cas12a-mCherry plasmids, respectively, then cloned into the pBlue-AAVS1-Puro-Cas9:p300-M2rtTA-AAVS1 plasmid [47] to replace Cas9:p300, next, two AAVS1 gRNA targeting sequences were inserted to flank the whole knock-in fragment.

For the plasmid pEF1α-CasRx-mCherry, the CasRx sequence was amplified from plasmid EF1a-CasRx-2A-EGFP (addgene, #109049), and used to replace *Lb*Cas12a sequence in plasmid pCAG-*Lb*Cas12a-mCherry.

For the luciferase reporter, the Tet operator containing six gRNA binding sites was amplified from the plasmid pBlue-AAVS1-Puro-Cas9:p300-M2rtTA-AAVS1 and the sequence of luciferase-polyA was synthesized, and then the two sequences were sub-cloned into the pBluSKM vector.

For the *Kras*^*G12D*^ donor, the *Lb*Cas12a gRNA targeting sequence within the *Rosa26* locus was synthesized and replaced the original sequence in the plasmid pBlue-Rosa26-IR-T2A-Puro- pEF1α-KrasG12D-IR-Rosa26 [42].

The plasmid pCAG-d*Lb*Cas12a-p300-mCherry, pCAG-d*Lb*Cas12a-VPR-mCherry, pCAG-d*Lb*Cas12a-10×Suntag-mCherry, pHRdSV40-scFv-GCN4-sfGFP-VP94-GB1-NLS, pHRdSV40-scFv-GCN4-sfGFP-VPR-GB1-NLS, pCAG-*Lb*Cas12a-mCherry, pCAG-*Lb*Cas12a-N57-mCherry, pCAG-d*As*Cas12a-VPR-mCherry, pCAG-*As*Cas12a-mCherry and pBlue-Rosa26-IR-T2A-Puro- pEF1α-KrasG12D-IR-Rosa26 were described previously [37, 42, 48, 49].

All sgRNAs were designed through https://benchling.com/ and ligated to the corresponding sgRNA expression plasmid. The sequences of all sgRNAs, linkers, and extended structures are listed in Additional file 2: Table S1. All constructs were verified through Sanger sequencing.

### Linker design

To increase editing efficiency, different linkers were designed by screening several digital libraries. Briefly, linker libraries were constructed with 5' and 3' linkers containing a 10-nt polyAC sequence and 5–7nt random bases, and the structure with each linker pair was predicted by RNAfold. The ones predicted to contain correct ribozyme structure and gRNA scaffold structure were selected out and ranked by the Gibbs free energy change (ΔG). The top 10–20 circular RNAs in the rank list were divided into groups according to structural similarity. The structure of group members was predicted by mFold, another RNA structure prediction tool. In each group, the circular RNA with the lowest ΔG (most stable) and correct structure predicted by both RNAfold and mFold was selected for the wet-experiment test.

### Cell culture and transfection

Cell lines were obtained from ATCC and regularly checked for mycoplasma. HEK293T and B16 cells were cultured in DMEM medium (Life Technologies), and MCF7 cells were maintained in RPMI 1640 medium (Life Technologies) at 37 °C under 5% CO_2_. All growth media were supplemented with 100 U/mL penicillin, 100 μg/mL streptomycin (Life Technologies), and 10% FBS.

For transient transfection experiments, plasmids were transfected into cells by polyethylenimine (PEI). Cells were plated in 24-well plates and transfected when reached about 70% confluence. For the mNeonGreen reporter HEK293T cells, 15.6.ng gRNA plasmid, 46.9 ng Cas12a / CasRx plasmid, and 62.5ng pBluSKM plasmid were mixed and co-transfected into cells to test the DNA or RNA cleavage of mNeonGreen. For the Tet-d*Lb*Cas12a-p300 knock-in HEK293T cells, 15.6ng gRNA plasmid and 484.4ng pBluSKM plasmid were mixed and co-transfected into cells to test target gene activation. For non-KI HEK293T or MCF7 cells, 15.6ng gRNA plasmid, 46.9ng *Lb*Cas12a based activator plasmid, and 437.5ng pBluSKM plasmid were mixed and co-transfected. For the Tet-*Lb*Cas12a knock-in HEK293T cells, 62.5ng gRNA plasmid and 437.5ng pBluSKM plasmid were mixed and co-transfected to test endogenous gene cleavage. For CasRx-mediated endogenous gene degradation, 62.5ng gRNA plasmid, 187.5ng CasRx plasmid, and 250ng pBluSKM plasmid were mixed and co-transfected into HEK293T cells or MCF7 cells. Two days (for CasRx) or 4 days (for Cas12a) after transfection, cells were harvested for gene expression analyses, flow cytometry, Deep-seq, Tag-seq, RNA-seq, or TIDE analyses etc.

The Tet-d*Lb*Cas12a-p300 and Tet-*Lb*Cas12a knock-in HEK293T cell lines were obtained by transfecting corresponding plasmids and selecting positive clones with 1μg/ ml puromycin and were added 2μg/ ml doxycycline to induce Cas protein expression.

For testing the stability of gRNAs, 5 μg/ ml actinomycin D was added to cells 24 h after transfections, then cells were harvested at indicated time points to analyze linear or circular gRNA content.

### Quantitative real-time PCR

Total RNA was extracted from cells using TRIzol reagent (Invitrogen) according to the manufacturer’s protocol. Briefly, the total RNA was extracted from cells by adding 500 μl TRIzol and 100μl chloroform, after centrifugation at 13,000 rpm for 10min at 4°C, the supernatant was transferred to a 1.5-mL RNase-free tube. RNA was purified by precipitation with isopropanol and 75% ethanol. Five hundred nanogram RNA was reverse transcribed using Prime Script™ RT Reagent Kit (TAKARA). cDNA was diluted 10-fold, and 2.0µl diluted cDNA was used for each RT-PCR reaction with TB Green Premix Ex Taq II Kit (TAKARA) and Lightcycler 96 (Roche). The primers are listed in Additional file 2: Table S1.

### RNA-seq

Gene activation of d*Lb*Cas12a-p300 activator and RNA cleavage of CasRx were determined by RNA-seq as described previously [48]. Briefly, total RNA was isolated from cells using TRIzol reagent (Invitrogen), purified by magnetic beads with Oligo(dT), and random fragmented by fragmentation buffer. The first strand was synthesized by six-base random hexamers, and then followed by the second strand synthesis. After purification, terminal repair, and dA-tailing addition, and adaptor addition, double-strand cDNA was amplified by PCR to complete library construction. After quality verified using Qubit 3.0, Agilent 2100 Bioanalyzer and agarose gel electrophoresis, libraries were sequenced by Illumina HiSeq instrument with a 150-bp paired-end module. Significant differentially expressed genes were defined with a false discovery rate (FDR) < 0.05 and a fold change > 2.

### Tag-seq

The detailed procedures for Tag-seq library construction and analyses were described in our previous work [37]. Briefly, genomic DNA (gDNA) was isolated from cells using TIANamp Genomic DNA Kit (TIANGEN). Then the genomic DNA was fragmented, end-repaired, and ligated by dA-tail. The library was constructed with Nested PCR and sequenced with Illumina HiSeq instrument with a 150-bp paired-end module. The sequencing data were analyzed through Tag filtering, quality control, read alignment, PCR duplicate consolidation, and identification of RGN-mediated off-target cleavage sites. The Tag-seq data analysis pipeline is available at GitHub (https://github.com/zhoujj2013/Tag-seq).

### Deep-seq

Multiplexed DNA cleavage efficiency of *Lb*Cas12a was determined by Deep-seq as described previously [48]. Briefly, the primers with forward and reverse indexes were used to amplify on-target sites in the first-round PCR. Then, equal amounts of the first PCR products were mixed for a second round PCR with the P5- and P7-containing primers to generate the libraries. The library was sequenced by Illumina HiSeq instrument with 150-bp paired-end reads. Pooled samples were demultiplexed by the indexes within the primers for the first-round PCR. Sequencing reads were trimmed, mapped to, and aligned with the genome reference. Indels were called using the R package Genomic Alignments [50]. The primers are listed in Additional file 2: Table S1.

### FACS analysis

In the mNeonGreen reporter assay, cells were harvested 2 or 4 days after transfection and resuspended in 400 μl FACS buffer (1× DPBS, 0.2% BSA, 2mM EDTA), then loaded onto a flow cytometer (BD Fortessa, CA, USA) to detect mNeonGreen- and mCherry-positive cells. The cleavage efficiency of Cas12a or CasRx was calculated as the proportion of mNeonGreen-negative cells and the mean fluorescence intensity (MFI) of mNeonGreen within the transfected cells (mCherry positive). For the collateral effects assay of CasRx, MFI of mCherry and mNeonGreen of total cells were analyzed. For examining the cleavage efficiency of CasRx when targeting endogenous genes, transfected cells (mCherry positive) were sorted out for RNA extraction by MoFlo XDP flow cytometry sorter 48h after transfection.

### DFHBI-1T staining and microscopy

Cells were observed 2 days after transfection. Thirty minutes before imaging, the culture medium was changed to FluoroBrite medium (ThermoFisher) with 40 μM DFHBI-1T and 0.1 μg/ml Hoechst. Live cell fluorescence images were acquired on a Nikon microscope.

Western blotting

Cells were lysed in 2X SDS loading buffer (200 mM β-mercaptoethanol, 100 mM Tris pH 6.8, 20% glycerol, 4% SDS, 0.05% bromophenol blue). The lysates were separated by SDS-PAGE and transferred onto the NC membrane, followed by blocking with 5% milk in TBST solution and incubation with primary antibody overnight at 4 °C and secondary antibodies for 1h at room temperature. Finally, the NC membrane was incubated with Immobilon Western Chemiluminescent HRP Substrate (Millipore) and imaged by Gel Imager System. Antibodies included Anti-HA-tag antibody (MBL, M180-3) for d*Lb*Cas12a-p300, d*Lb*Cas12a-VPR, d*Lb*Cas12a-SunTag, scFv-sfGFP-VP64, scFv-sfGFP-VPR, *Lb*Cas12a, d*As*Cas12a-VPR, *As*Cas12a, and CasRx. Anti-Tubulin antibody (Proteintech, 66240), GAPDH antibody (Proteintech, 60004), and HRP-conjugated horse anti-mouse IgG secondary antibody (CST 7076S).

### H&E staining and immunohistochemistry

Samples were fixed overnight in 4% paraformaldehyde at 4°C, embedded with paraffin, and then sliced into 5-μm sections. For H&E staining, the sections were rehydrated with gradient ethanol and stained with hematoxylin and eosin. For immunohistochemistry, the rehydrated sections were boiled for 15 min to retrieve antigen. After the endogenous peroxidase was blocked for 15 min, the sections were sequentially incubated with a primary antibody (Anti-CK19, ab133496, Abcam) overnight at 4 °C, a secondary antibody (HRP-conjugated anti-rabbit IgG secondary antibody, 7074S, CST) for 1h at room temperature, and the chromogenic substrate for 20 min. Finally, the sections were counterstained with hematoxylin, dehydrated, and sealed with neutral resins.

### Mice

C57BL/6 female mice were purchased from Guangdong Animal Center. In the luciferase reporter assay, each mouse aged 6 weeks was injected with a total of 26 μg plasmids (10 μg luciferase reporter plasmid, 12 μg pCAG-d*Lb*Cas12a-VPR-mCherry plasmid, and 4 μg sgRNA plasmid were mixed in 1.8-2.0 ml 0.9% sterile saline) via hydrodynamic tail vein injection (HTVI). Fifteen minutes before imaging, each group which included three mice injected with different gRNAs was treated with 200 μl 15mg/ml D-luciferin potassium salt (Beyotime) and then imaged with an exposure time of 10 s. In the cancer modeling assay, each mouse aged 6 weeks was injected with a total of 55 μg plasmids (30 μg *Kras*^*G12D*^ donor plasmid, 20 μg pCAG-*Lb*Cas12a-N57-mCherry plasmid, and 5 μg sgRNA plasmid were mixed in 1.8–2.0 ml 0.9% sterile saline) via hydrodynamic tail vein injection. After 7 weeks, the mice were sacrificed for assessment. The livers were harvested for weighing, genomic DNA extraction, H&E staining, and immunohistochemistry.

## Supplementary Information

**Additional file 1:** **Fig. S1 **Establishment of the d*Lb*Cas12a-p300knock-in HEK293T cell line. **Fig. S2 **Circular gRNAs increase thetranscription efficiency of *Lb*Cas12a-based activators.** Fig. S3 **Establishment of the *Lb*Cas12a knock-in HEK293Tcell line and off-target analysis of *Lb*Cas12a with different gRNAs.** Fig. S4 **Circular gRNAs improve the DNA cleavage efficiency of *Lb*Cas12ain MCF7 cells. **Fig. S5 **comparison between cgRNA with other extendedstructuralized gRNAs. **Fig. S6 **Circular gRNAs improve the gene expressionor DNA cleavage of *As*Cas12a-based effectors. **Fig. S7 **Multiplexedgene activation and cleavage guided by cgRNAs. **Fig. S8 **Efficient andspecific RNA cleavage activity of CasRX with cgRNA. **Fig. S9 **Thetrans-cleavage activity of CasRx–mediated exogenous transcripts degradation. **Fig.S10 **The trans-cleavage activity of CasRx–mediated endogenous transcriptsdegradation. **Fig. S11 **The structures of circular gRNAs for *Lb*Cas12awith different linkers targeting *IL1RN* predicted by mFold. **Fig. S12**The structures of circular gRNAs for *Lb*Cas12a with different linkerstargeting *IL1RN* predicted by mFold. **Fig. S13 **The structures ofcircular gRNAs for CasRx with different linkers targeting *STAT3*predicted by mFold.

**Additional file 2:** **Table S1** The sequences of sgRNAs, primers and linkers.

**Additional file 3.** Peer review history.

## Data Availability

All sgRNAs, linkers, and primer sequences in this study are available in the Additional file 2: Table S1. RNA-seq, Tag-seq, and Deep-seq data have been deposited on the National Center for Biotechnology Information database (accession nos. PRJNA830337 [51]). All code for RNA-seq analysis pipeline in this study is available at https://github.com/YuchenLiu1621/circular_RNA and https://doi.org/10.5281/zenodo.7991279 [52]. All code for Deep-seq analysis pipeline in this study is available at https://github.com/TZH0511/deepseq and https://doi.org/10.5281/zenodo.7992742 [53]. All code for Tag-seq analysis pipeline in this study is available at https://github.com/zhoujj2013/Tag-seq and https://doi.org/10.5281/zenodo.4679460 [54]. Any updates will also be published on Zenodo and GitHub. All uncropped versions of the gel and microscopy images are available in FigShare (https://doi.org/10.6084/m9.figshare.23268527 [55]).
