# Supplementary material for: Engineered circular guide RNAs boost CRISPR/Cas12a- and CRISPR/Cas13d-based DNA and RNA editing
Source: Genome Biol. 2023 Jun 23;24:145. doi: 10.1186/s13059-023-02992-z (PMC10288759; doi:10.1186/s13059-023-02992-z)
Supplement: Supplementary file 1 — Additional file 1: Fig. S1 Establishment of the dLbCas12a-p300knock-in HEK293T cell line. Fig. S2 Circular gRNAs increase thetranscription efficiency of LbCas12a-based activators. Fig. S3 Establishment of the LbCas12a knock-in HEK293Tcell line and off-target analysis of LbCas12a with different gRNAs. Fig. S4 Circular gRNAs improve the DNA cleavage efficiency of LbCas12ain MCF7 cells. Fig. S5 comparison between cgRNA with other extendedstructuralized gRNAs. Fig. S6 Circular gRNAs improve the gene expressionor DNA cleavage of AsCas12a-based effectors. Fig. S7 Multiplexedgene activation and cleavage guided by cgRNAs. Fig. S8 Efficient andspecific RNA cleavage activity of CasRX with cgRNA. Fig. S9 Thetrans-cleavage activity of CasRx–mediated exogenous transcripts degradation. Fig.S10 The trans-cleavage activity of CasRx–mediated endogenous transcriptsdegradation. Fig. S11 The structures of circular gRNAs for LbCas12awith different linkers targeting IL1RN predicted by mFold. Fig. S12The structures of circular gRNAs for LbCas12a with different linkerstargeting IL1RN predicted by mFold. Fig. S13 The structures ofcircular gRNAs for CasRx with different linkers targeting STAT3predicted by mFold. [file 13059_2023_2992_MOESM1_ESM.zip › Additional file 1-Supplementary Figure1.pdf]

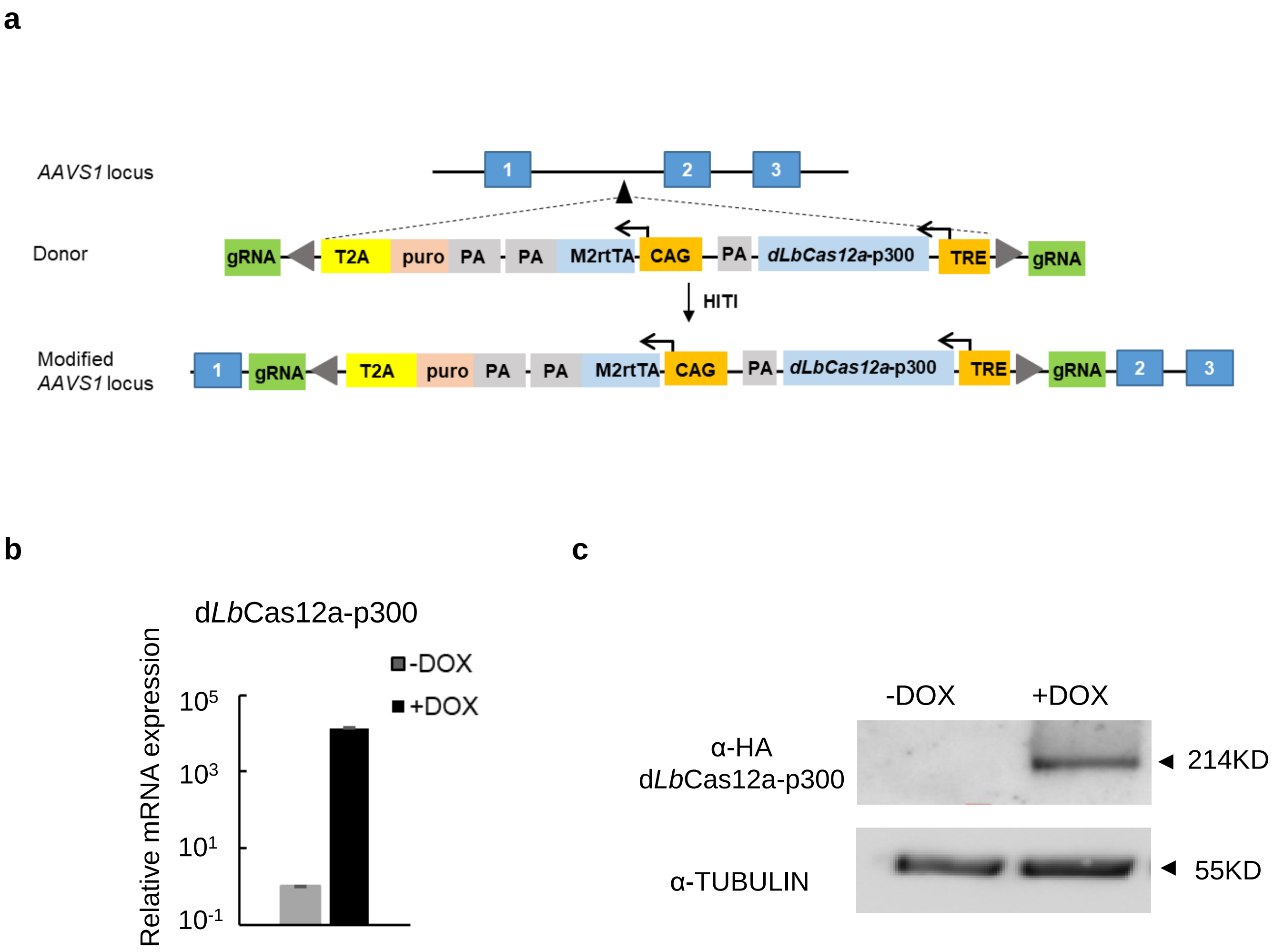

Fig. S1 Establishment of the d*Lb*Cas12a-p300 knock-in HEK293T cell line.

Fig. S1 Establishment of the d*Lb*Cas12a-p300 knock-in HEK293T cell line. a, Schematic of the DNA donor and targeting strategy at the *AAVS1* locus. Filled boxes with numbers indicate exons. gRNA indicates the gAAVS1 target site in the donor. T2A, *Thoseaasigna* virus 2A self-cleaving peptide; puro, puromycin resistant gene; PA, polyA; M2rtTA, reverse tetracycline-controlled transactivator; CAG, CAG promoter; d*Lb*Cas12a-p300, nuclease-deficient *Lb*Cas12a fused with the p300 core domain; TRE, TRE promoter containing tetracycline-responsive element; HITI, Homology Independent Target Integration. **b** & **c**, The inducible expression of d*Lb*Cas12a-p300 in the KI cell line. The cells were treated with or without 2ug/ml Doxycycline (Dox) for 48 hrs, RNA and protein were harvested for quantitative RT-PCR (**b**) and Western Blotting (**c**) assays, respectively.

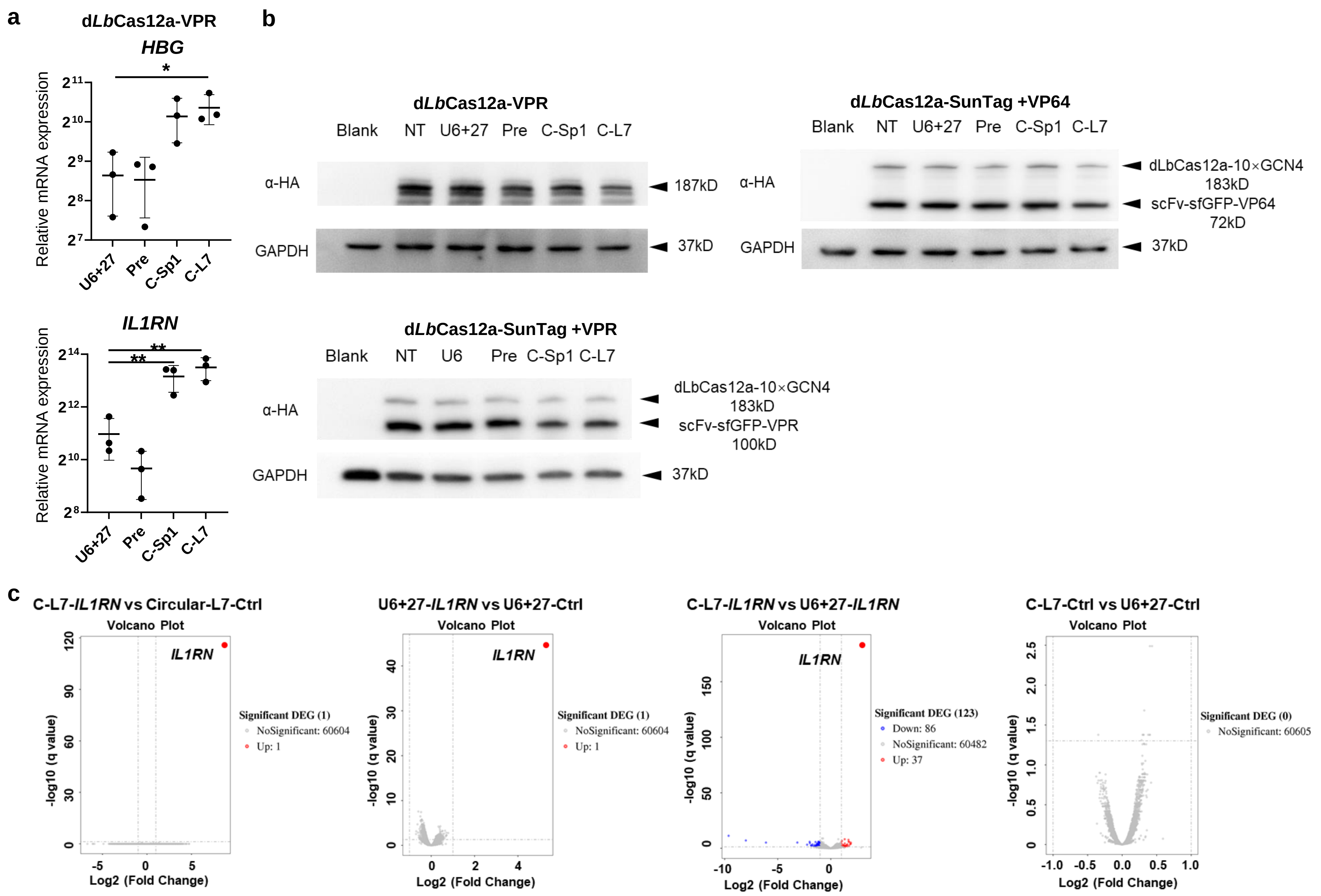

Fig. S2 Circular gRNAs increase the transcription efficiency of *Lb*Cas12a-based activators.

Fig. S2 Circular gRNAs increase the transcription efficiency of *Lb*Cas12a-based activators. **a**, cgRNA-directed gene activation in MCF7 cells. **b**, Western blot of *Lb*Cas12a-based activator proteins in transfected HEK293T cells. **c**, The specificity of cgRNA-directed gene activation showed with volcano plot. DEG, Differentially Expressed Genes. q <0.05, Fold change >2. For **a**, quantitative RT-PCR revealed relative mRNA expression of *IL1RN* and *HBG*. Mean values are presented with S.D., n = 3 independent experiments. For each experiment, fold changes of mRNA expression in tested samples versus that in the U6 + 27 linear mNeonGreen gRNA were shown. *p <0.05, **p <0.01, ***p <0.001, one-way ANOVA test.

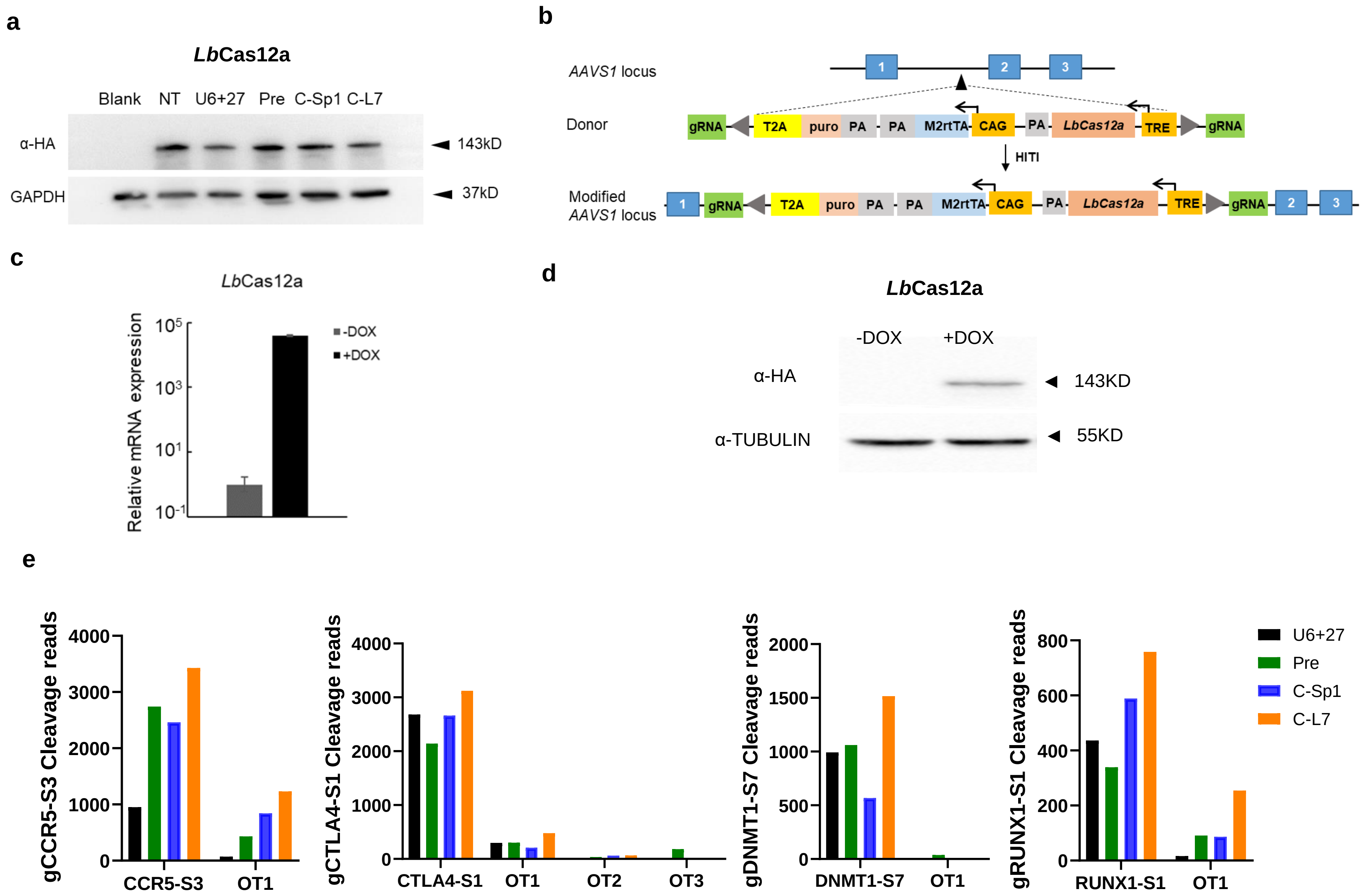

Fig. S3 Establishment of the *Lb*Cas12a knock-in HEK293T cell line and off-target analysis of *Lb*Cas12a with different gRNAs.

Fig. S3 Establishment of the *Lb*Cas12a knock-in HEK293T cell line and off-target analysis of *Lb*Cas12a with different gRNAs. a, Western blot of *Lb*Cas12a proteins in transfected mNeonGreen reporter cells. **b**, Schematic of the DNA donor and targeting strategy at the *AAVS1* locus. The strategy is as same as the d*Lb*Cas12a-p300 KI cell line except replacing the coding sequence of d*Lb*Cas12a-p300 with the one of *Lb*Cas12a. **c** & **d**, The inducible expression of *Lb*Cas12a in the KI cell line was confirmed by quantitative RT-PCR and Western Blotting**. e**, The sequencing read counts of off-target sites by Tag-seq. The *Lb*Cas12a knock-in HEK293T cells transfected with 14 gRNAs, and genomic DNA was extracted after 4 days post transfection. OT, off-target.

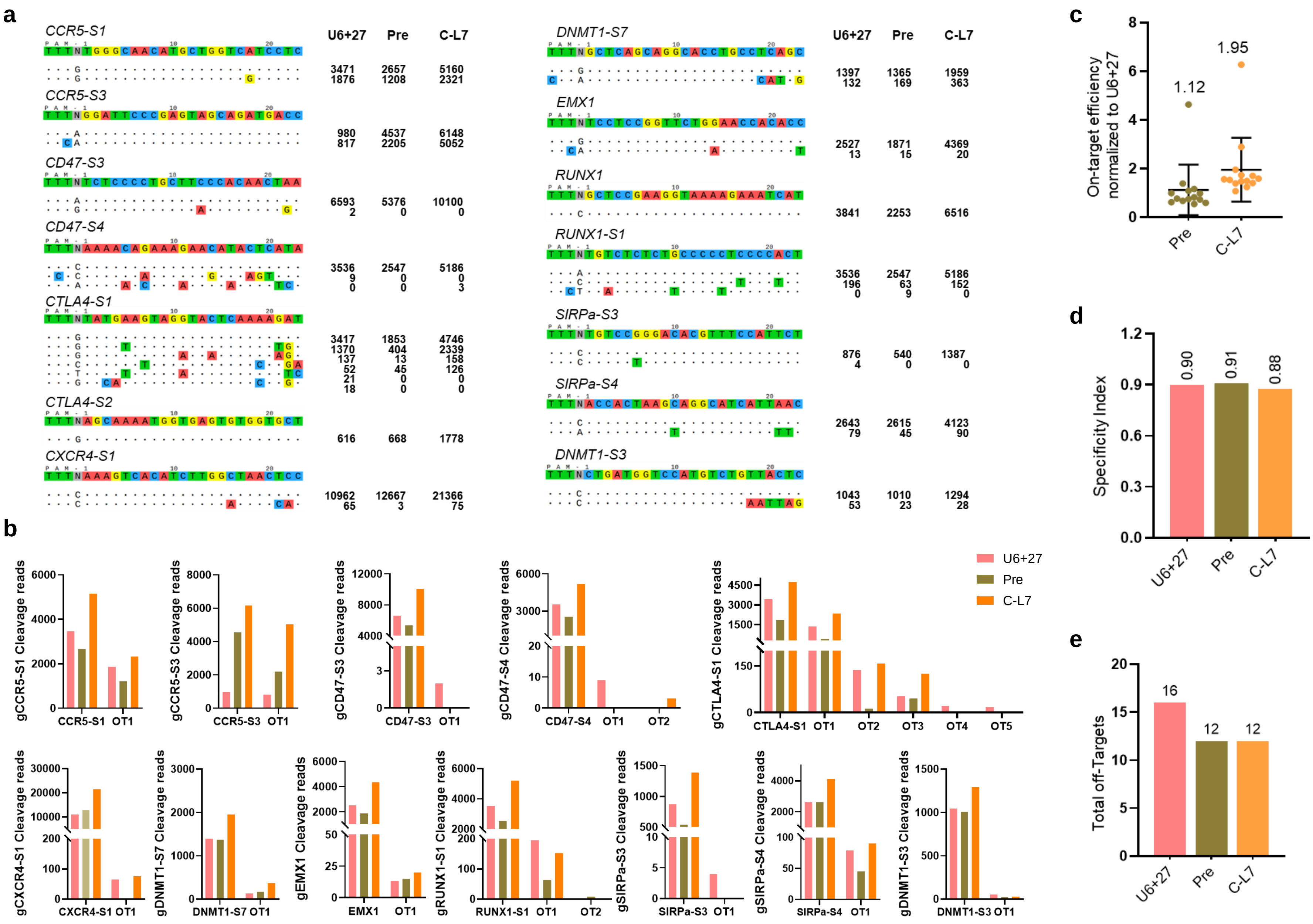

Fig. S4 Circular gRNAs improve the DNA cleavage efficiency of *Lb*Cas12a in MCF7 cells.

Fig. S4 Circular gRNAs improve the DNA cleavage efficiency of *Lb*Cas12a in MCF7 cells. a, Editing reads at the on-target and off-target sites revealed by Tag-seq in MCF7 cells targeting as the same 14 sites as in HEK293T cells. **b,** The sequencing read counts of off-target sites by Tag-seq. OT, off-target. **c,** Efficiency comparison between different gRNAs. **d**, Global on-target cleavage specificity assessment. **e**, Total number of off-target sites detected for the 14 sites. For **a-e**, MCF7 cells were co-transfected with plasmids encoding 14 gRNAs in the form of U6 + 27, or Pre, or C-L7 and *Lb*Cas12a, and 4 days later, the genomic DNA was harvested for Tag-seq analysis.

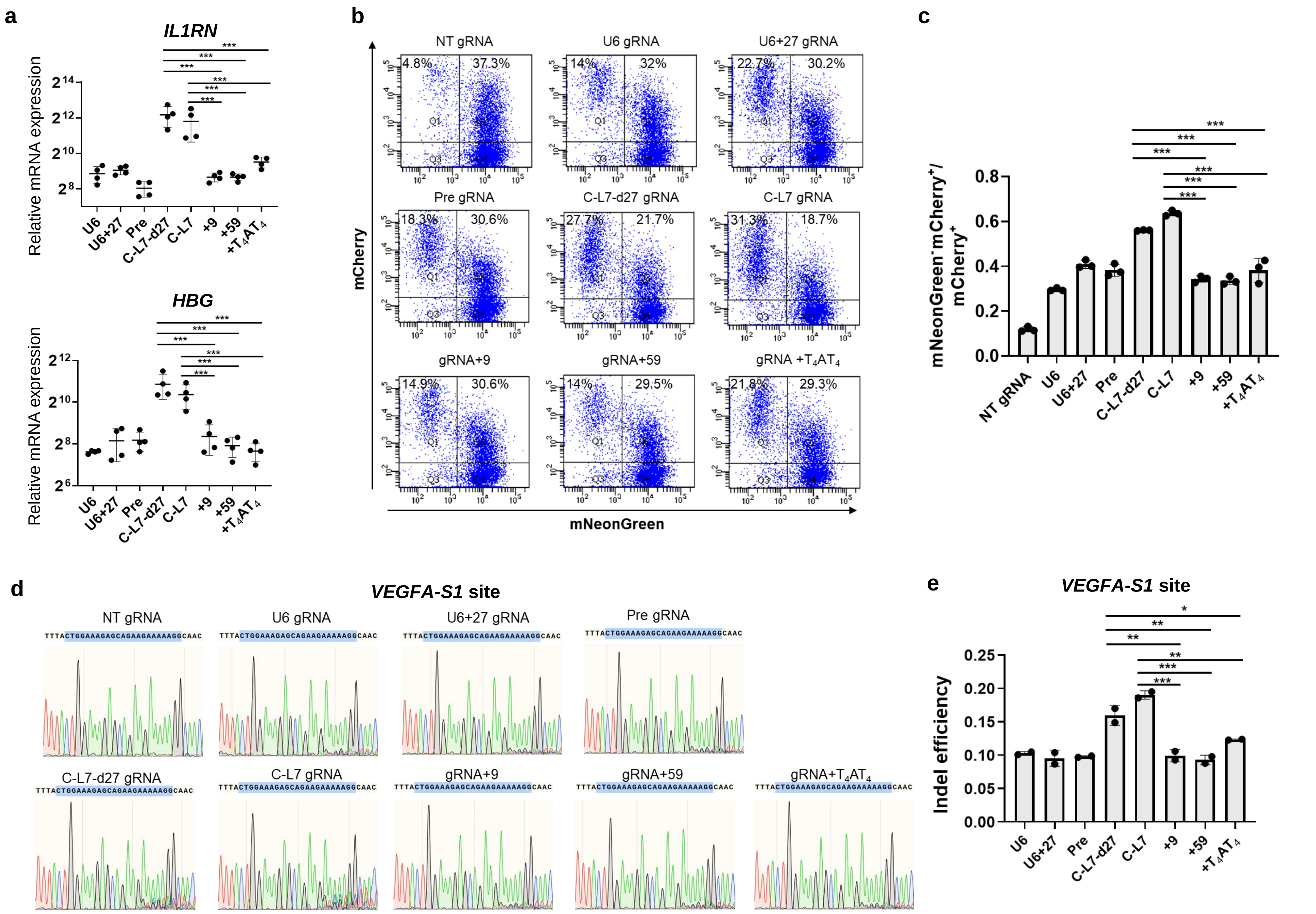

Fig. S5 comparison between cgRNA with other extended structuralized gRNAs.

Fig. S5 Comparison between cgRNA with other extended structuralized gRNAs. a, Gene activation guided by different extended structuralized gRNAs in the d*Lb*Cas12a-p300 knock-in HEK293T cell line. U6 + 27, the mature gRNAs driven by the polymerase III promoter U6 + 27 cassette; U6, the mature gRNAs driven by the polymerase III promoter U6; Pre, spacer flanked by two scaffold sequences and driven by the polymerase III promoter U6 + 27 cassette; C-L7-d27, circular gRNA with linker7 driven by the polymerase III promoter U6; C-L7, circular gRNA with linker7 driven by the polymerase III promoter U6 + 27 cassette; gRNA + 9, extending the 5' end of the crRNA with 9 nucleotides; gRNA + 59, extending the 5' end of the crRNA with 59 nucleotides; gRNA + T_4AT_4, extending the 3' end of the crRNA with TTTTATTTT sequences; n=4. **b-c**, FACS analyses of the mNeonGreen reporter cells 4 days after co-transfection with *Lb*Cas12a-P2A-mCherry and mNeonGreen-targeting-gRNA plasmids. The cleavage efficiency was quantified by the cell ratio of $mNeonGreen^{-}$ $mCherry^{+}$ / $mCherry^{+}$ in the FACS assays, n=3. **d-e**, Editing efficiency of the endogenous site VEGFA. The *Lb*Cas12a knock-in HEK293T cells were transfected with different extended structuralized gRNAs, and 4 days after transfection, genome DNA was harvested and analyzed by Sanger sequencing and Tracking of indels by decomposition (TIDE) assay, n=2. **p* <0.05, ***p* <0.01, ****p* <0.001, one-way ANOVA test.

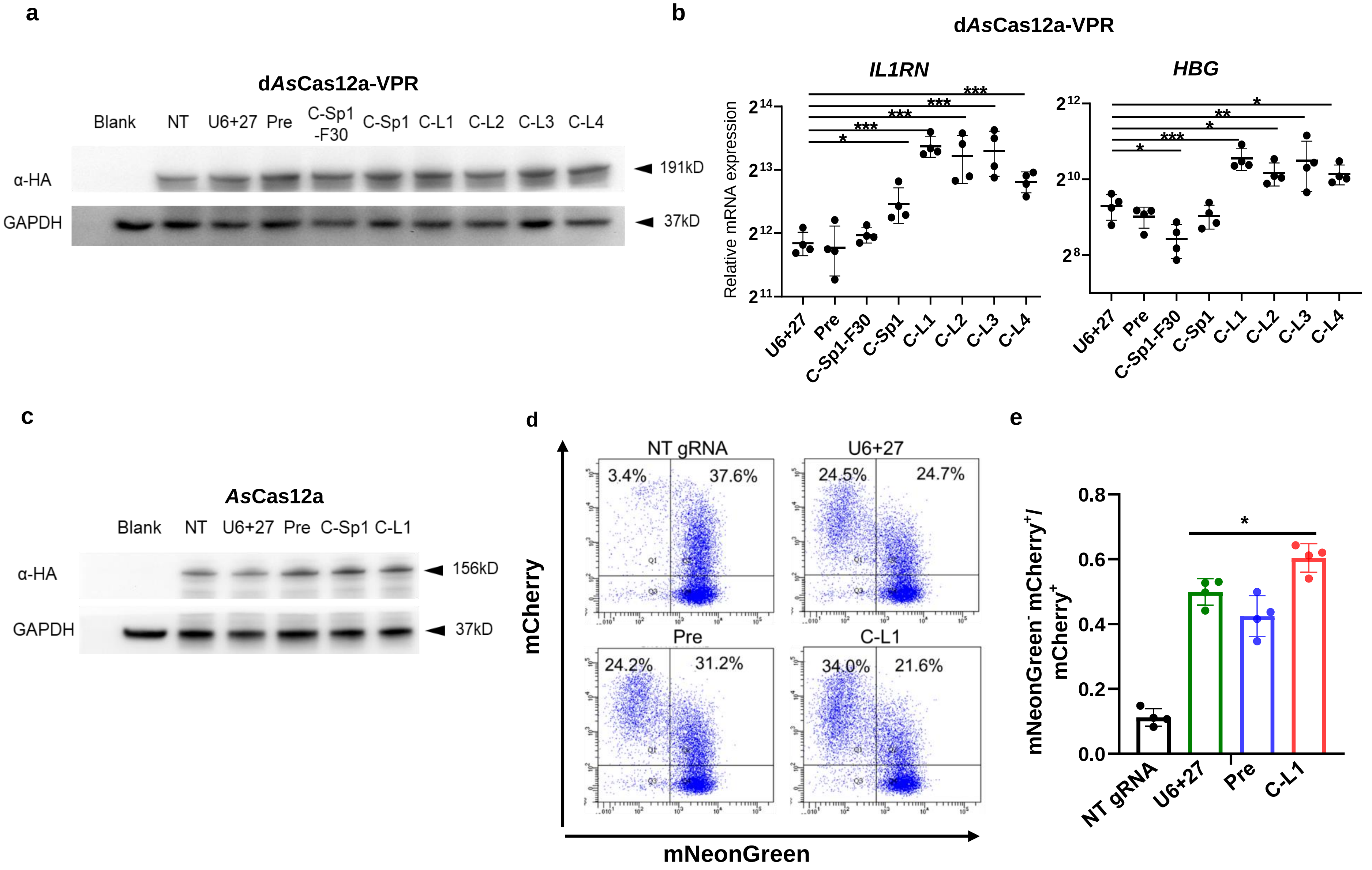

Fig. S6 Circular gRNAs improve the gene expression or DNA cleavage of *As*Cas12a-based effectors.

Fig. S6 Circular gRNAs improve the gene expression or DNA cleavage of *As*Cas12a-based effectors. a-b, Gene activation guided by cgRNAs with different linkers in HEK293T cells co-transfected with d*As*Cas12a-VPR and indicated gRNAs. Western blot of d*As*Cas12a-VPR proteins in transfected HEK293T cells (**a**). Quantitative RT-PCR revealed relative mRNA expression of *IL1RN* and *HBG* (**b**). **c-e**, FACS analyses of the mNeonGreen reporter cells 4 days after co-transfection with *As*Cas12a-P2A-mCherry and mNeonGreen-targeting-gRNA plasmids. Western blot of *As*Cas12a proteins in transfected mNeonGreen reporter cells (**c**). FACS analyses showed the cleavage of target gene mNeonGreen mediated by different gRNAs (**d**). The cleavage efficiency was quantified by the cell ratio of mNeonGreen^{-} mCherry^{+} / mCherry^{+} in the FACS assays (**e**). For **b, e**, n = 4 independent experiments. *p <0.05, **p <0.01, ***p <0.001, one-way ANOVA test.

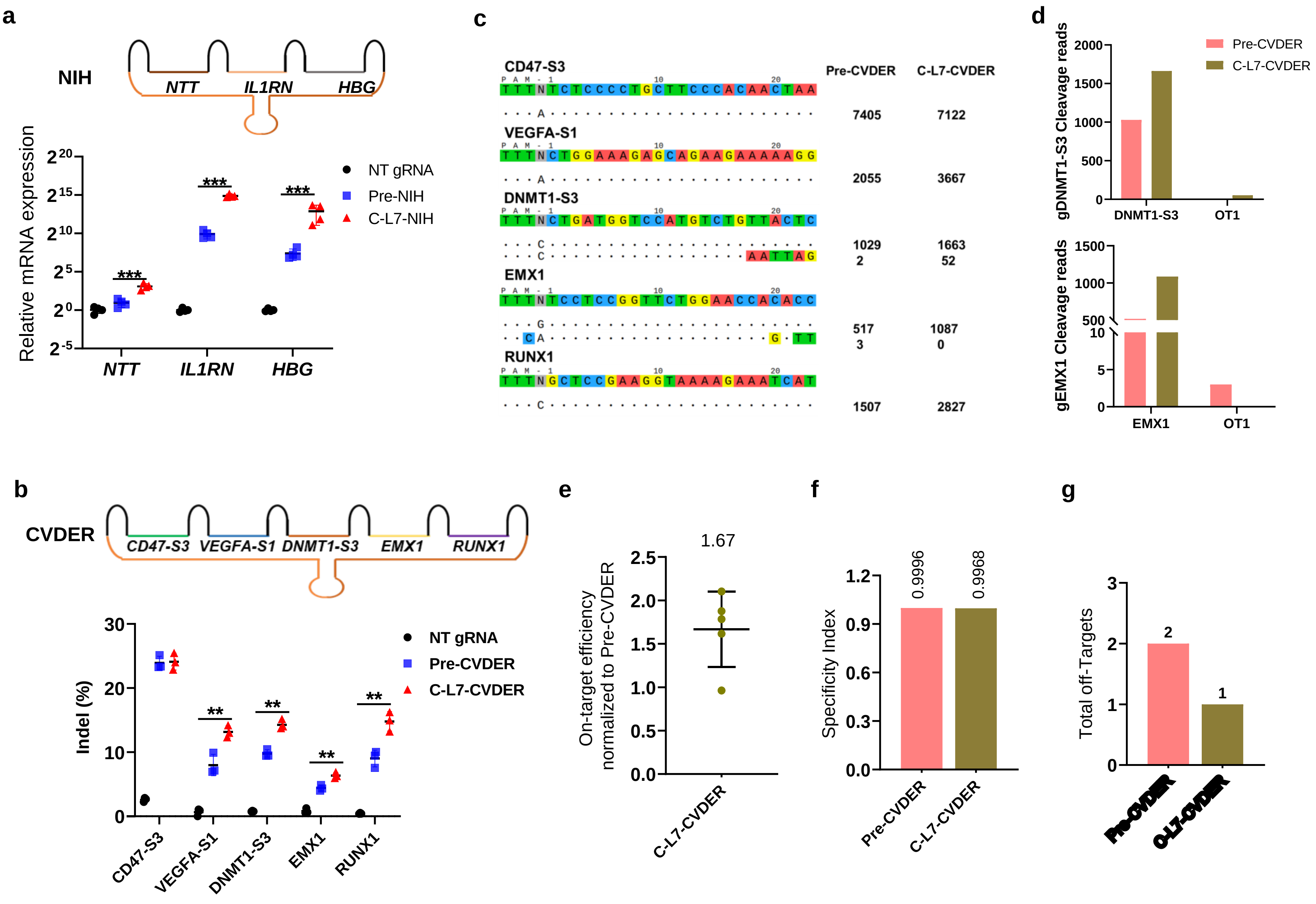

Fig. S7 Multiplexed gene activation and cleavage guided by cgRNAs.

Fig. S7 Multiplexed gene activation and cleavage guided by cgRNAs. a, Multiplexed gene activation guided by cgRNAs in the d*Lb*Cas12a-p300 KI HEK293T cells, n=4. **b,** Deep-seq shows multiplexed gene cleavage guided by cgRNAs in HEK293T cells, n=3**. c-g,** tag-seq shows multiplexed gene cleavage guided by cgRNAs in the KI HEK293T cells. **p <0.01, ***p <0.001, Student's t-test.

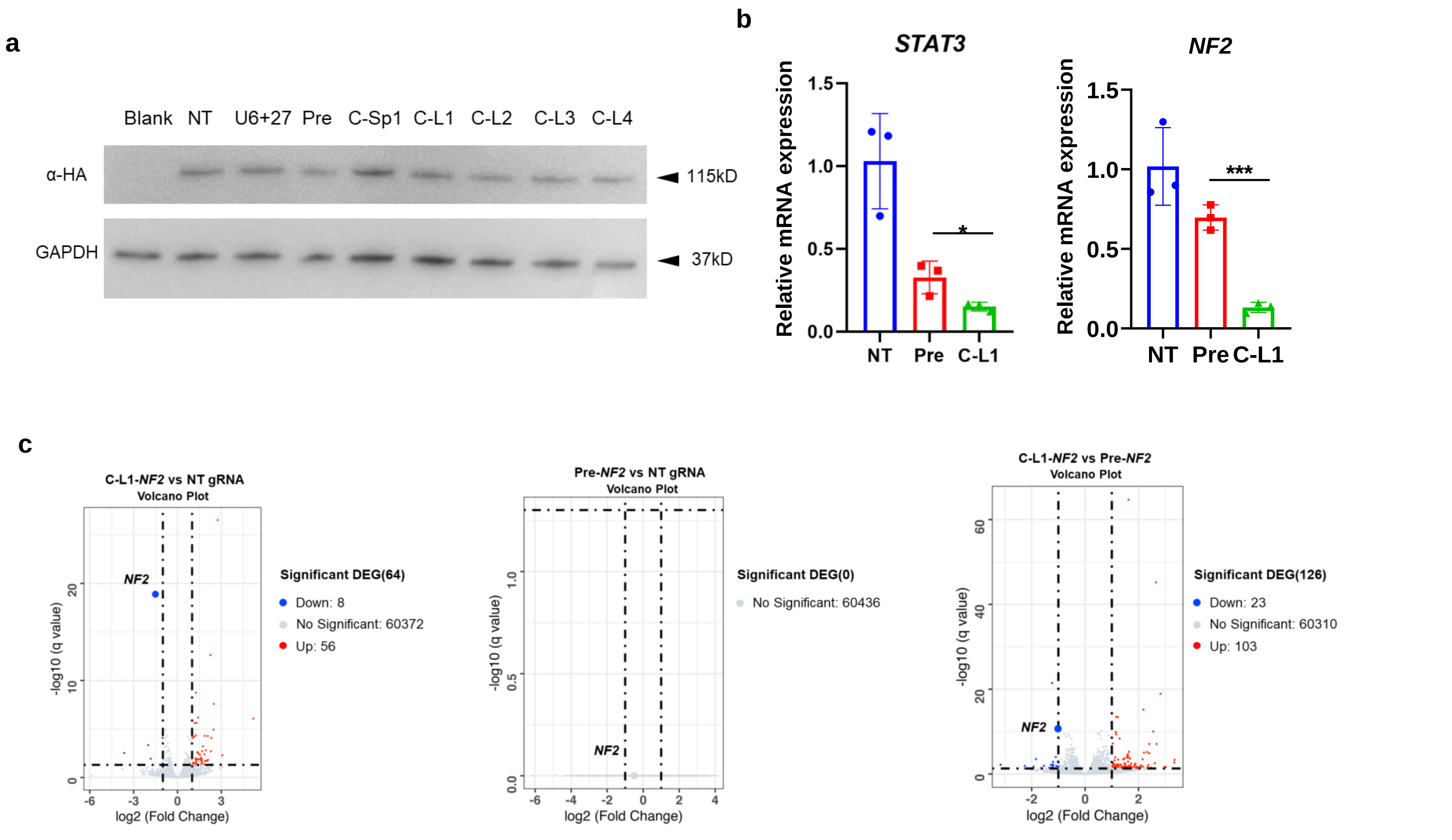

Fig. S8 Efficient and specific RNA cleavage activity of CasRX with cgRNA.

Fig. S8 Efficient and specific RNA cleavage of CasRX with cgRNA. a, Western blot of CasRx proteins in the transfected mNeonGreen reporter cells. b, The RNA cleavage efficiency of CasRx on endogenous genes in MCF Cells. **c**, The specificity of cgRNA-directed gene repression showed with volcano plot. q <0.05, Fold change >2. *p <0.05, ***p <0.001, Student's t-test.

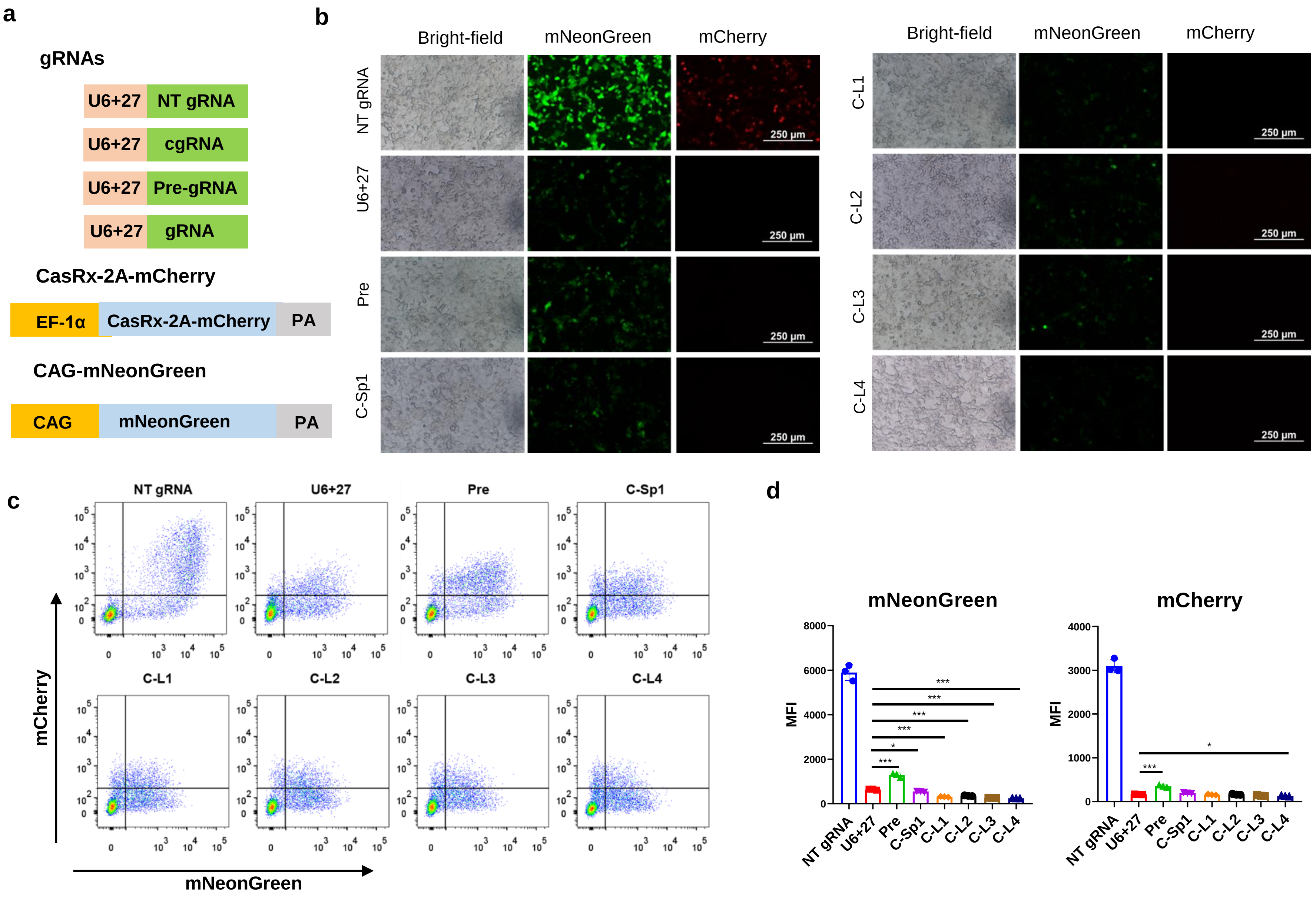

Fig. S9 The trans-cleavage activity of CasRx–mediated exogenous transcripts degradation.

Fig. S9 The trans-cleavage activity of CasRx–mediated exogenous transcripts degradation a, Experiment design for trans-cleavage activity of CasRx. HEK293T cells were transiently co-transfected with 500ng plasmids (75ng CasRx-P2A-mCherry, 25ng mNeonGreen-targeting-gRNA and 400ng CAG-mNeonGreen). The expression levels of mNeonGreen and mcherry were measured using FACS and fluorescence image 48 hrs after transfection, and the cleavage efficiency and trans-cleavage activity was quantified by the mean fluorescence intensity of mNeonGreen and mCherry respectively. **b,** Representative bright field and fluorescence images of HEK293T cells. Scale bar, 250 μm. **c,** Representative flow cytometry analyses of mNeonGreen and mCherry in HEK293T cells. **d,** FACS quantitative analysis of mean fluorescence intensity (MFI) of total cells. n=3, *p <0.05, ***p <0.001, one-way ANOVA test.

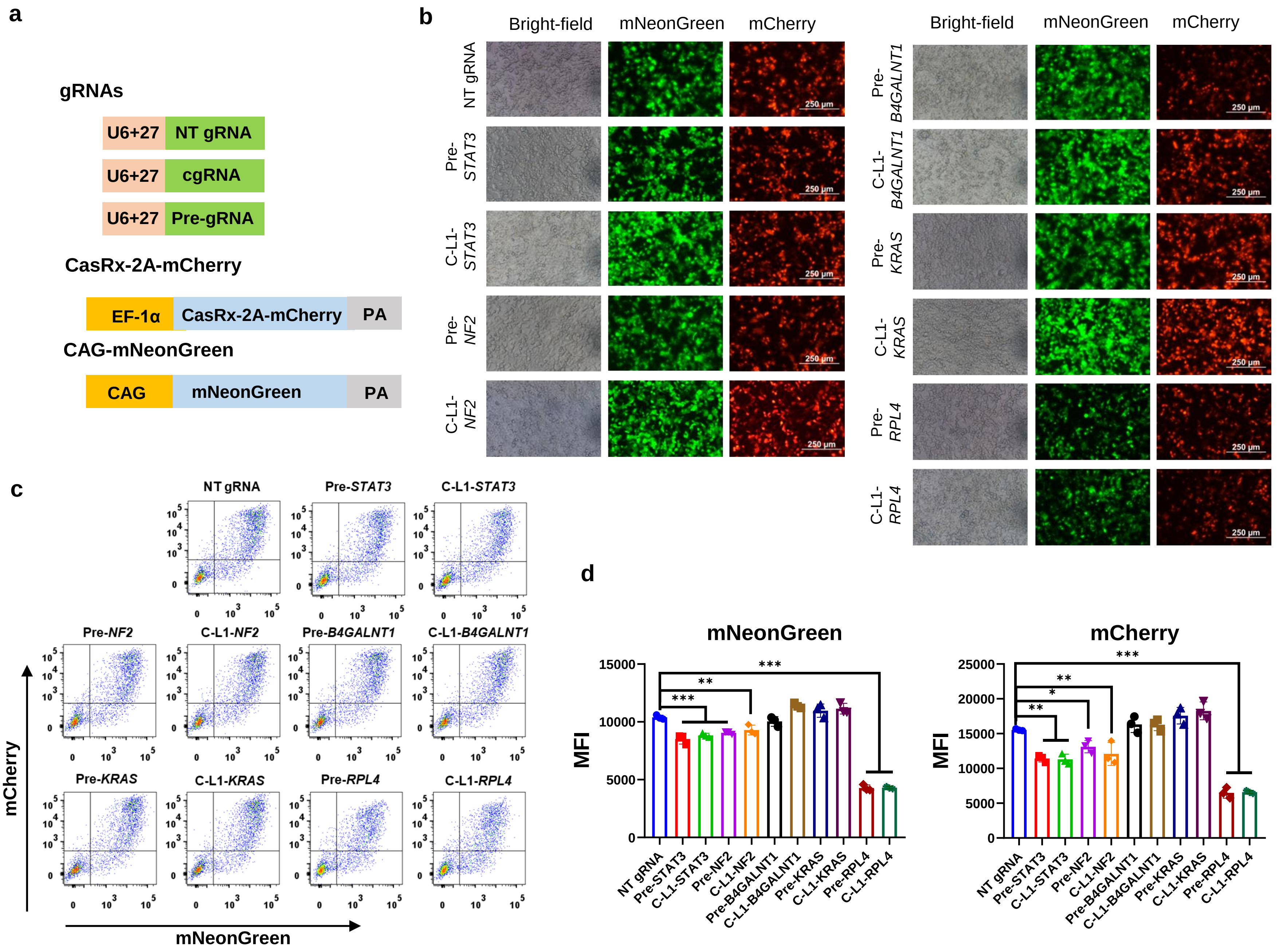

Fig. S10 The trans-cleavage activity of CasRx–mediated endogenous transcripts degradation.

Fig. S10 The trans-cleavage activity of CasRx–mediated endogenous transcripts degradation. a, Experiment design for trans-cleavage activity of CasRx. HEK293T cells were transiently co-transfected with CasRx-P2A-mCherry, CAG-mNeonGreen and endogenous-targeting-gRNA plasmids (gSTAT3, gNF2, gB4GALNT1, gKRAS, and gRPL4), The trans-cleavage activity was quantified by the fluorescence intensity of mNeonGreen and mCherry. **b,** Representative bright field and fluorescence images of the transfected HEK293T cells. Scale bar, 250 μm. **c-d**, Flow cytometry analyses of mNeonGreen and mCherry in the transfected HEK293T cells. n=3, *p <0.05, ***p <0.001, one-way ANOVA test.

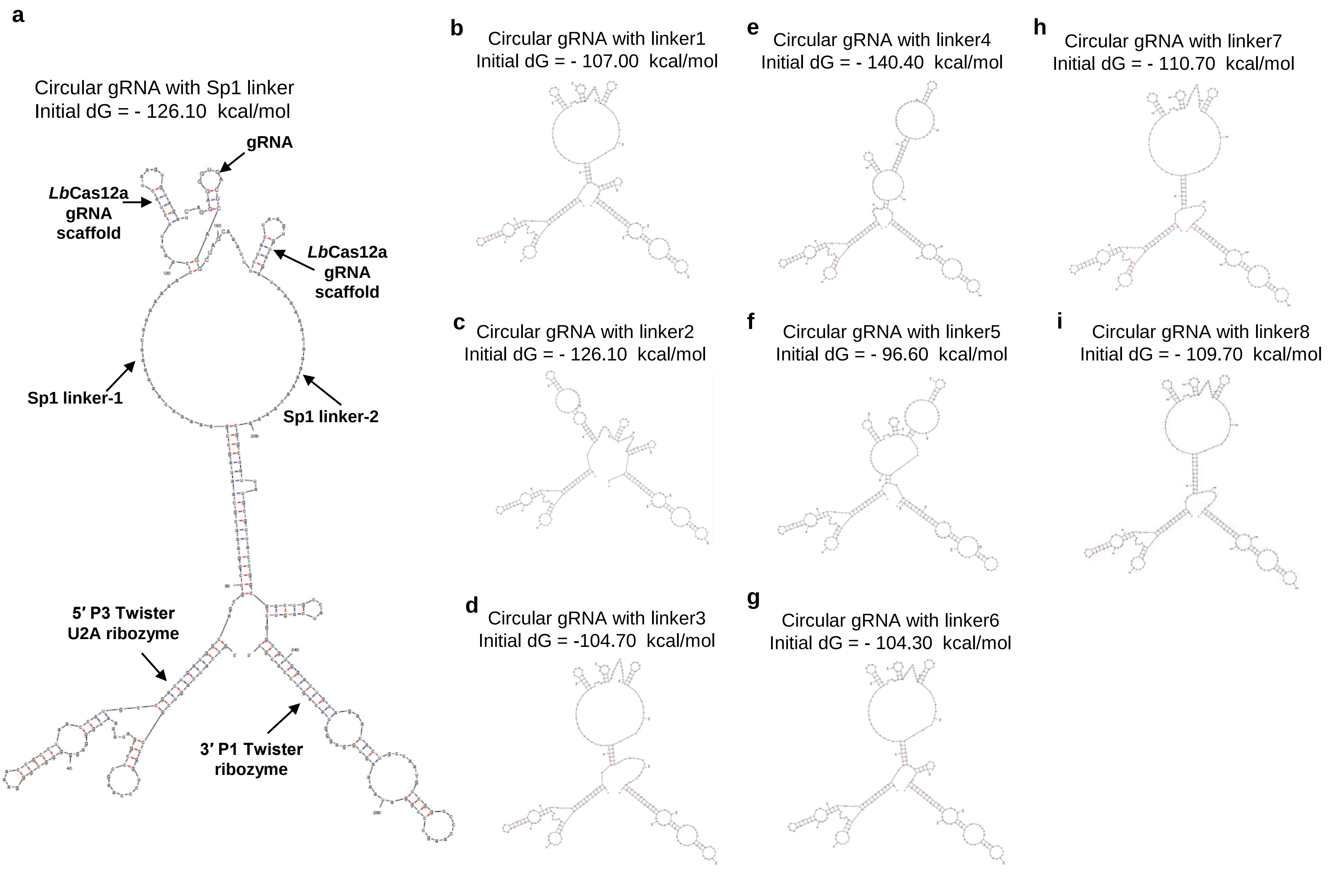

Fig. S11 The structures of circular gRNAs for *Lb*Cas12a with different linkers targeting *IL1RN* predicted by mFold.

Fig. S11 The structures of circular gRNAs for *Lb*Cas12a with different linkers targeting *IL1RN* predicted by mFold.

a-g, The predicted structure of circular gRNAs for *Lb*Cas12a with Sp1 linker, linker1, linker2, linker3, linker4, linker5, linker6, linker7 and linker8.

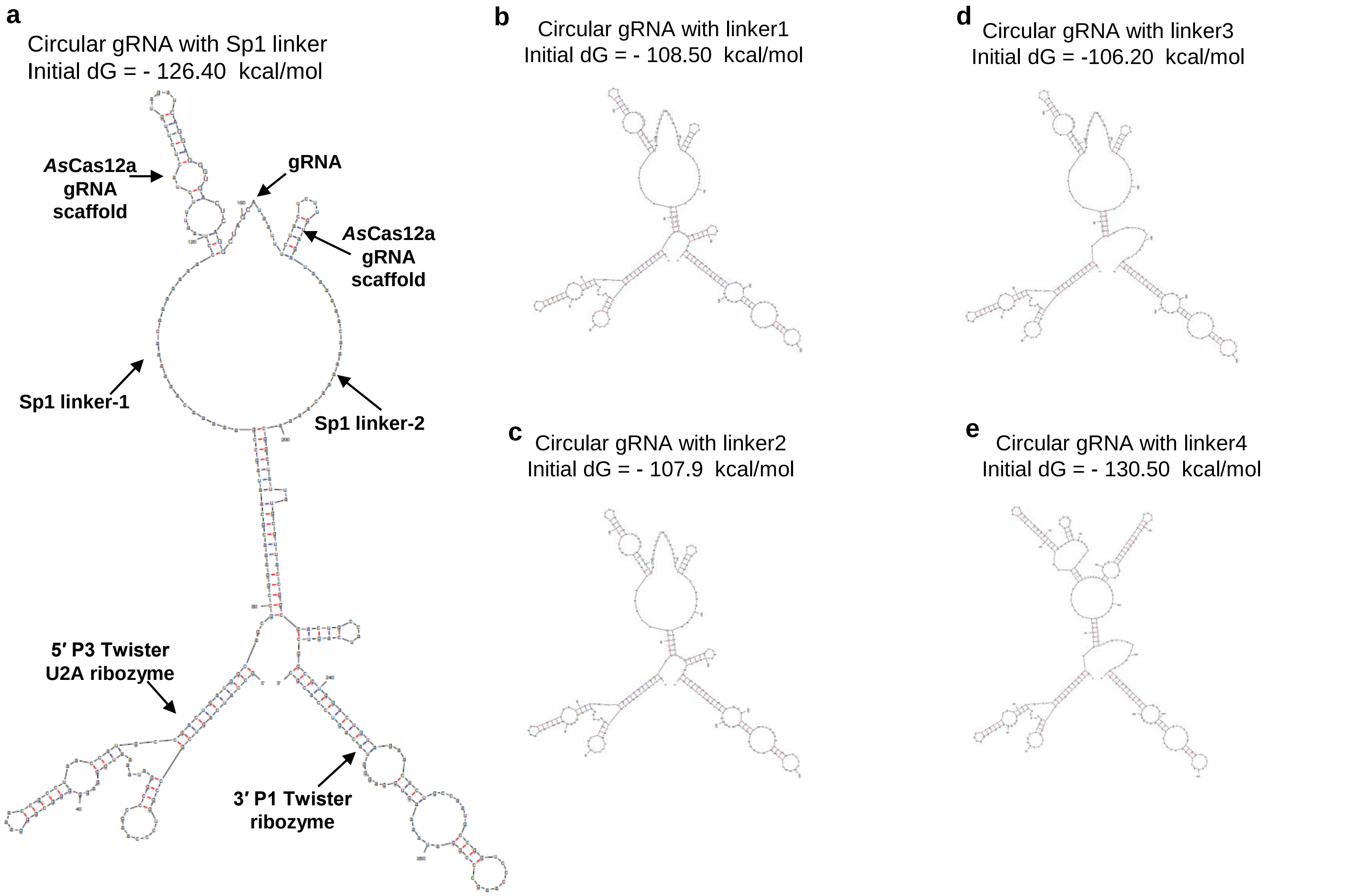

Fig. S12 The structures of circular gRNAs for *As*Cas12a with different linkers targeting *IL1RN* predicted by mFold.

Fig. S12 The structures of circular gRNAs for *As*Cas12a with different linkers targeting *IL1RN* predicted by mFold.

a-g, The predicted structure of circular gRNAs for *As*Cas12a with Sp1 linker, linker1, linker2, linker3 and linker4.

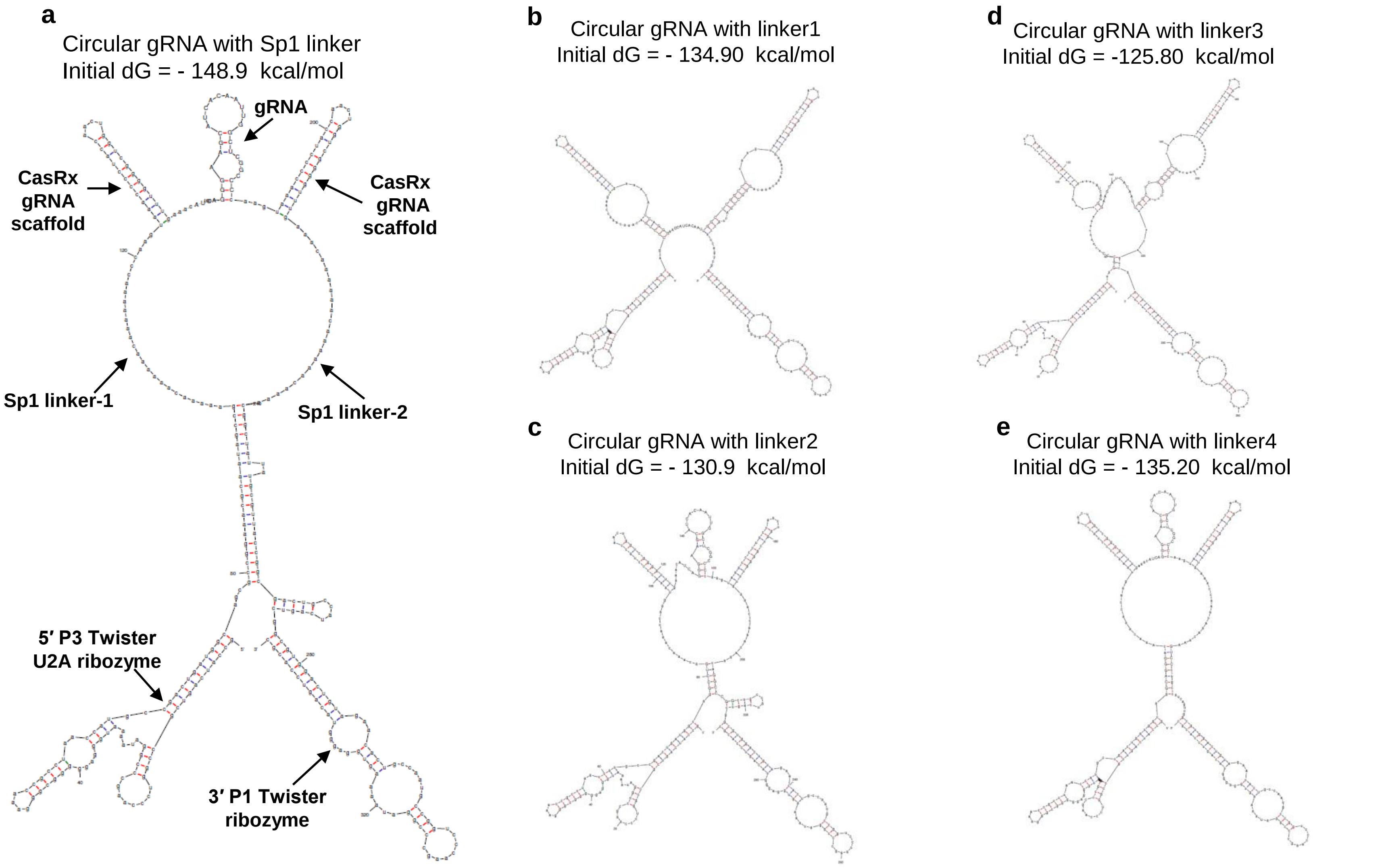

Fig. S13 The structures of circular gRNAs for CasRx with different linkers targeting *STAT3* predicted by mFold.

Fig. S12 The structures of circular gRNAs for CasRx with different linkers targeting *STAT3* predicted by mFold.

a-g, The predicted structure of circular gRNAs for CasRx with Sp1 linker, linker1, linker2, linker3 and linker4.
