# Supplementary material for: Engineered circular guide RNAs boost CRISPR/Cas12a- and CRISPR/Cas13d-based DNA and RNA editing
Source: Genome Biol. 2023 Jun 23;24:145. doi: 10.1186/s13059-023-02992-z (PMC10288759; doi:10.1186/s13059-023-02992-z)
Supplement: Supplementary file 3 — Additional file 3. Peer review history. [file 13059_2023_2992_MOESM3_ESM.docx]

**Review history**

**First round of review**

**Reviewer 1**

In this manuscript, the authors engineered circular gRNAs (cgRNAs) to improve the editing activity of CRISPR-Cas proteins. The cgRNAs have been proved to be more stable than conventional linear gRNAs. The use of cgRNAs increased the transcriptional activation and DNA cleavage efficiency of Cas12a and the RNA interference efficiency of CasRx.

Major issues:
1. Cas12a with high specificity and CasRx without altering the genome could play important roles in gene therapy and biotechnology applications in the future. However, in vivo application of them is hindered by the potential existence of collateral effects. Therefore, the effect of highly stable cgRNAs on trans-cleavage activity needs to be further verified.
2. Unlike the Cas12 DNA editing tools, CasRx enables gene manipulation at the transcriptional level. CasRx can down-regulate the gene expression levels instead of knocking out genes. Therefore, the quantitative criterion for cleavage efficiency of mNeonGreen is not sufficiently rigorous.
3. The detailed experiments need to be clearly described in Methods. Some abbreviations are never defined anywhere (e.g. RT-PCR, KI, DEG, FACS).

Minor issues:
1. In Fig1c, 1d, the Robozyme should be Ribozyme.
2. In Extended Data Fig. 5c, C-L7 should be C-L1.
3. "Similar to the in vitro results, cgRNA outperformed linear gRNA at each time point (Fig. 9b, c)." Fig. 9 can't be found in the manuscript.
4. Figure 4a, c showed that C-L4 is more efficient than C-L1. Why choose C-L1 for subsequent experiments?
5. "C-L1 cgRNAs were also observed to repress their expression more potently than the linear counterpart Pre gRNAs (Fig. 3c)." Fig. 3c should be Fig. 4c.

**Reviewer 2**

The author showed enhanced gene activation and RNA cleavage through circle gRNAs in the CRISPR/Cas12a and CRISPR/Cas13d systems. The current manuscript included both in vitro and in vivo data on cgRNA. The overall data is clearly arranged and convincing. However, some additional improvements may help strengthen this strategy.
1, In terms of specificity, despite the specificity was showed in Fig 3f, we suggest a separate figure showing the off-target reads be placed in either the main figure or supplementary figure. Indeed, the specificity was lower in cgRNAs, which should be described clearly and accurately.
2, Expression of Cas protein should be checked with linear or circular gRNAs.
3, What about the gRNA and Cas abundance in vivo? In fig.6C the result of C-L7-gRNA was not as stable as pre-gRNA, why?
4, Extended structuralized gRNA may help stabilize the gRNAs, can authors make comparisons between this strategy and cgRNA.
5, What about the off-targets of CasRX with cgRNA?
6, In the result sections, the author should describe detail important data instead of using "increase", "higher", "improved"……

**Authors’ response to reviewers**

Reviewer #1: In this manuscript, the authors engineered circular gRNAs (cgRNAs) to improve the editing activity of CRISPR-Cas proteins. The cgRNAs have been proved to be more stable than conventional linear gRNAs. The use of cgRNAs increased the transcriptional activation and DNA cleavage efficiency of Cas12a and the RNA interference efficiency of CasRx.

We thank the reviewer for the positive feedback on our manuscript.

Major issues:

1. Cas12a with high specificity and CasRx without altering the genome could play important roles in gene therapy and biotechnology applications in the future. However, in vivo application of them is hindered by the potential existence of collateral effects. Therefore, the effect of highly stable cgRNAs on trans-cleavage activity needs to be further verified.

We thank the reviewer for the instructive suggestion. We did observe collateral effects when using a high dose of the CasRx/sgRNA system to interfere RNA expression. To mitigate collateral effects, we transfected the mNeonGreen reporter cells with a low dose of the system (125 ng plasmid for one well of 24-well plate, including 46.8 ng CasRx-P2A- mCherry, 15.7 ng mNeonGreen-targeting-gRNA, and 62.5 ng pBluSKM blank plasmid) and observed mild collateral effects on mCherry expression (Fig. 4a).

Consistent with the previous report[1], we also observed obvious trans-cleavage activity of CasRx on mCherry expression when targeting mNeonGreen upon transient transfection of plasmids with a high dose (500 ng plasmid for one well of 24-well plate, including 75 ng CasRx-P2A-mCherry, 25 ng mNeonGreen-targeting-gRNA, and 400 ng mNeonGreen expression plasmid). As shown in Additional file 1: Fig. S9, HEK293T cells were transfected with the indicated plasmids, and 48 hours later, the expression levels of mNeonGreen and mCherry were measured using fluorescence imaging and fluorescence- activated cell sorting (FACS) analyses. The cleavage efficiency and trans-cleavage activity was quantified by the fluorescence intensity of mNeonGreen and mCherry, respectively.

As expected, cgRNAs enhanced CasRx-mediated mNeonGreen fluorescence decrease. In addition, we also observed significant reduction in mCherry fluorescence compared with the non-targeting gRNA control, suggesting substantial collateral effects of CasRx- mediated RNA degradation when targeting transiently overexpressed exogenous genes. Compared with linear U6 +27 gRNA, CgRNAs (Sp1, C-L1, C-L2, C-L3) showed a similar trans-cleavage activity, whereas C-L4 showed increased trans-cleavage activity compared to U6 +27 gRNA (Additional file 1: Fig. S9).

Next, to examine the trans-cleavage activity of CasRx when targeting endogenous genes, the plasmids EF1α-CasRx-P2A-mCherry, CAG-mNeonGreen and gRNAs targeting endogenous gene STAT3, NF2, B4GALNT1, KRAS or RPL4 (a target gene reported to induce dramatic collateral effects[1]) were co-transfected into HEK293T cells and the trans- cleavage activity was quantified by the fluorescence intensity of mNeonGreen and mCherry (Additional file 1: Fig. S10). Consistent with the previous report[1], RPL4 gRNA induced dramatic collateral effects. However, only slight collateral degradation was observed when targeting STAT3 and NF2, and no apparent collateral degradation was observed for B4GALNT1 and KRAS. More importantly, we observed no significant difference between cgRNAs and linear pre-gRNA (Additional file 1: Fig. S10).

The trans-cleavage activity of Cas12a were referred as RNA-guided DNA binding unleashes indiscriminate single-stranded DNA (ssDNA) cleavage activity, which was often used for nucleic acid detection[2]. When used for genome editing in vivo, target-activated Cas12a has the risk to cleave transiently exposed ssDNA during replication, transcription and homology-directed repair processes. However, Cas12a only recognizes two sites of a target gene in the genome of a diploid mammalian cell, and thus in theory, only two molecules of Cas12a/gRNA complex can be activated to cleave ssDNA, and protective DNA repairing mechanism can repair ssDNA cleavage even if ssDNA is cleaved by chance. Therefore, in principle, Cas12a would not cause broad ssDNA cleavage. And in fact, no collateral effects of Cas12a was observed in bacteria or in mouse embryos[3, 4].

In summary, for the collateral effects of CasRx, cgRNA was not observed to enhance the side effects. It is currently known that high expression of target RNA could activate more

CasRx to induce severe collateral effects, some unknown properties of gRNA might regulate trans-cleavage activity and thus different gRNAs targeting the same gene could induce different levels of collateral effects[1, 5]. CasRx variant with minimal collateral effects has been developed[1]. Our results also showed that delivering less CasRx would mitigate trans-cleavage effects (Fig. 4a & Additional file 1: Fig. S9). Therefore, by using the engineered CasRx variant, minimizing the delivered CasRX system, and screening out optimal gRNA would decrease the collateral effects. For the collateral effects of Cas12a, it has been demonstrated to be negligible for in vivo applications. Accordingly, we have revised our manuscript, please refer to the new version.

2. Unlike the Cas12 DNA editing tools, CasRx enables gene manipulation at the transcriptional level. CasRx can down-regulate the gene expression levels instead of knocking out genes. Therefore, the quantitative criterion for cleavage efficiency of mNeonGreen is not sufficiently rigorous.

We thank the reviewer for the helpful suggestion. Following the suggestion, we have used the mean fluorescence intensity of mNeonGreen in the transfected cells (mCherry positive) in the FACS assay to quantify protein expression levels and RT-PCR assay to quantify mRNA expression levels, and both analyses show that circular gRNAs are more efficient than the linear one to down-regulate the expression of mNeonGreen in the CasRx system (Fig. 4b & c).

3. The detailed experiments need to be clearly described in Methods. Some abbreviations are never defined anywhere (e.g. RT-PCR, KI, DEG, FACS).

We thank the reviewer for the critical and helpful comments. We apologized for these mistakes. Now we have revised the Methods section and defined the abbreviations in the new manuscript. Please refer to the revised manuscript.

Minor issues:

1. In Fig1c, 1d, the Robozyme should be Ribozyme.

We thank the reviewer for pointing out the typo. And we have corrected it in the new

manuscript.

2. In Extended Data Fig. 5c, C-L7 should be C-L1.

We thank the reviewer for pointing out the typo. And we have corrected it in the new manuscript.

3. "Similar to the in vitro results, cgRNA outperformed linear gRNA at each time point (Fig. 9b, c)." Fig. 9 can't be found in the manuscript.

We thank the reviewer for pointing out the typo. Fig.9 should be Fig. 5, and we have corrected it in the new manuscript.

4. Figure 4a, c showed that C-L4 is more efficient than C-L1. Why choose C-L1 for subsequent experiments?

We thank the reviewer for the critical and helpful comment. We agree that the cleavage efficiency of C-L4 tends to be improved compared to C-L1, but using unpaired t-test, we did not observe statistically significant difference between C-L4 and C-L1 to mediate mNeonGreen degradation. So, as C-L1 is the No.1 cgRNA, we choose it for subsequent experiments.

5. "C-L1 cgRNAs were also observed to repress their expression more potently than the linear counterpart Pre gRNAs (Fig. 3c)." Fig. 3c should be Fig. 4c.

We thank the reviewer for pointing out the typo. And we have corrected it in the new manuscript.

Reviewer #2: Please consider the following questions:

- Are the methods appropriate to the aims of the study, are they well described, and are necessary controls included? If not, please specify what is required. yes.
- Are the conclusions adequately supported by the data shown? If not, please explain yes.
- Are sufficient details provided to allow replication and comparison with related analyses

that may have been performed? If not, please specify what is required. See my questions.

- Does the work represent a significant advance over previously published studies? yes.
- Is the paper of broad interest to others in the field, or of outstanding interest to a broad audience of biologists? yes.

The author showed enhanced gene activation and RNA cleavage through circle gRNAs in the CRISPR/Cas12a and CRISPR/Cas13d systems. The current manuscript included both in vitro and in vivo data on cgRNA. The overall data is clearly arranged and convincing.

We thank the reviewer for the positive feedback on our manuscript.

However, some additional improvements may help strengthen this strategy.

1. In terms of specificity, despite the specificity was showed in Fig 3f, we suggest a separate figure showing the off-target reads be placed in either the main figure or supplementary figure. Indeed, the specificity was lower in cgRNAs, which should be described clearly and accurately.

We thank the reviewer for the critical and helpful comments. As suggested, we have added Additional file 1: Fig. S3e and S4b to show the specificity of LbCas12a in HEK293T cells and MCF7 cells, respectively. In addition, we have added Additional file 1: Fig. S7d to show the specificity of LbCas12a in multiplexed gene cleavage. As mentioned by the reviewer, cgRNAs showed a slightly lower specificity compared to the linear counterpart. And we have described this observation in the Results section in the revised manuscript. Please refer to the revised main-text.

2. Expression of Cas protein should be checked with linear or circular gRNAs.

We thank the reviewer for the helpful suggestion. For the dLbCas12a-p300 and LbCas12a knock-in HEK293T cell lines, the Cas protein expression was checked by Western blot with or without Doxycycline treatment for 48 hours (Additional file 1: Fig. S1c & S3d). For the transient transfection assays, the expression of Cas protein with linear or circular gRNAs

was also checked by Western blot as shown in Additional file 1: Fig. S2b, S3a, S6a, S6c, and S8a.

3. What about the gRNA and Cas abundance in vivo? In fig.6C the result of C-L7-gRNA was not as stable as pre-gRNA, why?

We thank the reviewer for the instructive suggestion.

For the DNA cleavage of Cas12a in vivo (Fig. 6), we delivered plasmids by hydrodynamic tail vein injection (HTVI), which could deliver nucleic acids to the liver via the rapid intravascular injection of a large volume of liquid. The plasmids were transiently delivered into the liver cells, but could induce permanent genome DNA cleavage and tumorigenesis. Therefore, 7 weeks after HTVI, all the plasmids had been clean out and no expression of gRNA or Cas protein could be detected.

The low DNA cleavage activity of Cas12a and mouse individual might result in the observation that C-L7-gRNA was not as stable as pre-gRNA. The editing activity of Cas12a is observed to be lower than Cas9[6]. Consistently, our previous work has shown that Cas9-N57 system could induce a number of large tumor nodules in the liver in 4 weeks[7], while LbCas12a-N57 system only induce a few small tumor nodules with Pre-gRNA in 7 weeks. Therefore, liver weight and surface tumor nodule number of the Pre-gRNA group show no statistical difference compared to the Non-target gRNA control group. C-L7 cgRNA induce more indels than the linear Pre gRNA, and thus generate more and larger tumor nodules. In the C-L7 cgRNA group, due to individual mouse differences, tumors in some mice have become large enough to affect liver weight, while tumor nodules in the other mice are only visible on the liver surface and are too small to affect liver weigh in 7 weeks. Therefore, liver weight and surface tumor numbers of the C-L7 cgRNA group showed obvious difference, and, in theory, the mice with small tumor nodules would affect liver weight when they are sacrificed at an extended time point.

4. Extended structuralized gRNA may help stabilize the gRNAs, can authors make comparisons between this strategy and cgRNA.

We thank the reviewer for the instructive suggestion. Following the suggestion, we have

made comparison between three extended structuralized gRNAs and cgRNA in gene activation and cleavage of LbCas12a (Additional file 1: Fig. S5).

First, the U6+27 expression cassette, containing the first 27 nucleotides of human U6 RNA, has been reported to be capped with γ-methyl phosphates and accumulate to higher level than U6 transcripts[8]. Thus, in fact, we have used U6+27 cassette to express the U6+27- gRNA, Pre-gRNA and cgRNA in this work. In order to test whether the U6+27 cassette improves the efficiency of gene editing in the CRISPR/LbCas12a system, we constructed the U6 cassette to express the linear and circular gRNAs. Gene activation assay of IL1RN and HBG and DNA cleavage assay of mNeonGreen reporter gene and endogenous gene VEGFA showed no statistically significant different between the U6+27 and the U6 cassettes (Additional file 1: Fig. S5).

Next, it has been reported that extending the 5′ end of the gRNA could enhance the gene editing efficiency of the Cas12a ribonucleoproteins complexes (RNP) by 9 nucleotides (gRNA + 9) and 59 nucleotides (gRNA + 59)[9, 10]. Therefore, we compared these two structured gRNAs with cgRNA. Reverse transcription PCR (RT-PCR) revealed that cgRNAs (C-L7-d27 and C-L7) more potently activated gene expression than the 5’ end extended gRNA (gRNA + 9 and gRNA + 59) with about 8.8-11.4-fold change in IL1RN activation and 4.9-6.3-fold change in HBG activation (Additional file 1: Fig. S5). In addition, FACS and TIDE analyses showed that cgRNAs outperformed the 5’ end extended gRNAs (+ 9 and + 59) for DNA cleavage (Additional file 1: Fig. S5).

Finally, the crRNA with a U4AU4 3′-overhang has been reported to be more favorable to bind Cas12a to improve editing activity[11]. Thus, we also compared this structured gRNA with cgRNA. As shown in Additional file 1: Fig. S5, cgRNAs (C-L7-d27 and C-L7) exhibited better efficiency than the 3’ end extended gRNA (gRNA + T4AT4) both in gene activation and DNA cleavage.

In summary, we have made comparison between cgRNA and 5’ and 3’ extended structuralized gRNAs in gene activation and cleavage of LbCas12a, and cgRNA outperforms all the tested extended gRNAs.

5, What about the off-targets of CasRX with cgRNA?

We thank the reviewer for the instructive suggestion. Following the suggestion, we have performed RNA-seq assay and found that NF2 (the target gene) was significantly decreased in the C-L1 cgRNA sample compared to the Pre and NT gRNA samples with 2.4-fold and 3.6-fold change, respectively (Fig. 4e; Additional file 1: Fig. S8c). Of note, 64 and 126 differentially expressed genes (DEGs) were observed when comparing the C-L1 cgRNA group to the NT gRNA group and to the Pre gRNA group, respectively, indicating nonspecific and collateral cutting as well as the associated secondary effects[1, 5, 12].

6, In the result sections, the author should describe detail important data instead of using "increase", "higher", "improved"……

We thank the reviewer for the helpful comments. And we have added statistical fold change values when describing important data. Please refer to the revised manuscript.

Reference:

1. Tong H, Huang J. High-fidelity Cas13 variants for targeted RNA degradation with minimal collateral effects**.** Nat Biotechnol. 2023; 41:108-119.
2. Chen JS, Ma E, Harrington LB, Da Costa M. CRISPR-Cas12a target binding unleashes indiscriminate single-stranded DNase activity**.** Science. 2018; 360:436-439.
3. Wei Y, Zhou Y, Liu Y, Ying W, Lv R, Zhao Q, Zhou H, Zuo E, Sun Y, Yang H, Zhou C. Indiscriminate ssDNA cleavage activity of CRISPR-Cas12a induces no detectable off- target effects in mouse embryos**.** Protein Cell. 2021; 12:741-745.
4. Marino ND, Pinilla-Redondo R, Bondy-Denomy J. CRISPR-Cas12a targeting of ssDNA plays no detectable role in immunity**.** Nucleic Acids Res. 2022; 50:6414-6422.
5. Shi P, Murphy MR, Aparicio AO, Kesner JS, Fang Z, Chen Z, Trehan A, Guo Y, Wu X. Collateral activity of the CRISPR/RfxCas13d system in human cells**.** Commun Biol. 2023; 6:334.
6. Huang H, Lv W, Li J, Huang G, Tan Z, Hu Y, Ma S, Zhang X, Huang L, Lin Y. Comparison of DNA targeting CRISPR editors in human cells**.** Cell Biosci. 2023; 13:11.
7. Ma S, Wang X, Hu Y, Lv J, Liu C, Liao K, Guo X, Wang D, Lin Y, Rong Z. Enhancing site- specific DNA integration by a Cas9 nuclease fused with a DNA donor-binding domain**.** Nucleic Acids Res. 2020; 48:10590-10601.
8. Good PD, Krikos AJ, Li SX, Bertrand E, Lee NS, Giver L, Ellington A, Zaia JA, Rossi JJ, Engelke DR. Expression of small, therapeutic RNAs in human cell nuclei**.** Gene Ther. 1997; 4:45-54.
9. Park HM, Liu H, Wu J, Chong A, Mackley V, Fellmann C. Extension of the crRNA enhances

Cpf1 gene editing in vitro and in vivo**.** Nat Commun. 2018; 9:3313.

10. Paul CP, Good PD, Winer I, Engelke DR. Effective expression of small interfering RNA in human cells**.** Nat Biotechnol. 2002; 20:505-508.
11. Bin Moon S, Lee JM, Kang JG, Lee NE, Ha DI, Kim DY, Kim SH, Yoo K, Kim D, Ko JH, Kim YS. Highly efficient genome editing by CRISPR-Cpf1 using CRISPR RNA with a uridinylate- rich 3'-overhang**.** Nat Commun. 2018; 9:3651.
12. Ai Y, Liang D, Wilusz JE. CRISPR/Cas13 effectors have differing extents of off-target effects that limit their utility in eukaryotic cells**.** Nucleic Acids Res. 2022; 50:e65.

**Second round of review**

**Reviewer 1**

The authors have addressed all my questions well.

**Reviewer 2**

They have addressed my questions.
